# Evaluation of Treatment Response and Survival Outcomes in Anaplastic Thyroid Cancer Patients Following Surgery With and Without Other Treatment Modalities: A Systematic Review

**DOI:** 10.1002/hsr2.70710

**Published:** 2025-04-30

**Authors:** Hossein Negahban, Nazila Heidari, Amirhossein Heidari, Yekta Ghane, Mohammad Shirkhoda, Amirmohsen Jalaeefar

**Affiliations:** ^1^ Cancer Research Center of Cancer institute Tehran University of Medical Sciences Tehran Iran; ^2^ School of Medicine Iran University of Medical Sciences Tehran Iran; ^3^ Research Center for Advanced Technologies in Cardiovascular Medicine, Cardiovascular Diseases, Research Institute Tehran University of Medical Sciences Tehran Iran; ^4^ Faculty of Medicine, Tehran Medical Sciences Islamic Azad University Tehran Iran; ^5^ School of Medicine Tehran University of Medical Sciences Tehran Iran; ^6^ Department of Surgical Oncology, Cancer Institute Tehran University of Medical Sciences Tehran Iran

**Keywords:** anaplastic thyroid cancer, surgery, systematic review, thyroid neoplasm, thyroidectomy

## Abstract

**Background and Aims:**

Anaplastic thyroid carcinoma (ATC) is a rare type of malignancy ranking among the most aggressive diseases globally, with an extremely poor prognosis. No optimal standardized treatment has been established yet to promote ATC's prognosis and increase the patients' median survival. We aim to assess the effectiveness of surgery alone or combined with other treatment approaches for ATC patients.

**Methods:**

PubMed, Web of Science, and Scopus databases were systematically searched until June 1st, 2023. Study selection was limited to English retrospective studies. A citation search was also performed for the final articles that were included. This study followed the Preferred Reporting Items for Systematic Reviews and Meta‐Analyses (PRISMA) guidelines for systematic reviews and meta‐analyses.

**Results:**

During our search, we came to include 56 articles containing 16,246 patients suffering from ATC. We assessed the overall survival by treatment method and stage, emphasizing surgery's role. The most common efficacious treatment option in patients with resectable cancer is a combination of surgery with adjuvant chemoradiotherapy. However, surgery for stage IVC patients is controversial. Additionally, surgery and multimodality treatment can be affected by patients' characteristics, such as tumor size.

**Conclusions:**

Stage IVA and IVB resectable cancers may benefit from the combination of surgery and adjuvant therapies. However, the effectiveness of invasive treatments and the selection of appropriate adjuvant therapy options for IVC‐stage patients are still controversial.

**Abbreviations**
ATC: Anaplastic thyroid carcinoma
ChT: Chemotherapy
CA4P: Combretastatin A‐4 phosphate
CRT: Chemoradiation therapy
EBRT: External beam radiation therapy
FDA: United States Federal Drug Agency
JAK: Janus kinase
MEK: Mitogen‐activated protein kinase
NCDB: National Cancer Database
OS: Overall survival
PDGRF: Platelet‐derived growth factor receptor
RT: Radiation therapy
TKI: Tyrosine kinase inhibitor
VEGRF: Vascular endothelial growth factor receptor

## 1 Introduction

Anaplastic thyroid carcinoma (ATC), also known as undifferentiated thyroid carcinoma, is an uncommon malignant carcinoma that represents 2 to 3 percent of all thyroid cancers and ranks among the most aggressive diseases in the world with an extremely poor prognosis [1]. ATC is more prevalent in areas of endemic goiter among elderly people in their sixth to seventh decades of life, with a female predominance (female‐to‐male ratio: 2:1) [1, 2]. ATC is most commonly characterized by thyroid masses (77%) and node masses (54%), dysphagia (40%), as well as neck pain (26%) in patients [3]. With an initial presentation featuring metastasis in more than 50% of cases, all ATC tumors are categorized as stage 4 cancers by the American Joint Committee on Cancer [4]. Despite multimodal management, these patients' median overall survival (OS) is reported to be three to ten months, with a median 1‐year survival of 20% and a median ten‐year OS of less than 2% [5].

Concerning treatment approaches, no standardized therapeutic option has been developed to improve the prognosis of ATC and increase the median survival of the patients [6]. In the past, ATC patients were treated with cytotoxic chemotherapy (ChT) and radiation therapy (RT), with or without surgery [7]. Despite this, a significant percentage of patients were referred to palliative/hospice care. Over the last decade, a great deal of progress has been made in the molecular profile of ATC, leading to advancements in targeted therapies and immunotherapies [5, 8, 9]. Currently, surgical and other multidisciplinary adjuvant therapies can be considered for patients with a targetable disease and a significant response to treatment [7].

Despite guidelines recommending surgery combined with chemoradiation for patients in IVA/B stages, as well as a palliative approach for IVC subjects, no definitive treatment has yet been identified [10]. Therefore, we aim to systematically evaluate surgery with and without other treatment modalities as well as assess the efficacy of these treatment options regarding increasing the median OS and quality of life in ATC patients.

## 2 Material and Methods

The current systematic review was carried out following the Preferred Reporting Items for Systematic Reviews and Meta‐Analyses (PRISMA) checklists [11]. PRISMA checklists for systematic reviews and abstracts are illustrated in the supplementary tables (Table S1 and Table S2, respectively).

**Table 1:** Characteristics, demographics, and overall survival of anaplastic thyroid carcinoma patients among the included studies.

Study ID (Auhtor, country, year)	Study Type	Number of patients	Sex ratio (F: Female)	Mean age at diagnosis, year (Range or mean ± SD)	Stage	Cancer information (size, metastasis)	Type of treatment	Overall survival	Treatment response	Safety and adverse events
Jacob, Germany, 2023 [13]	Single‐Center retrospective cohort	63	F: 59%	68 (38–88)	IVA (2%), IVB (54%), IVC (44%)	LN involvement (*n* = 40) Distant metastases: at the time of diagnosis (44%), in the course of treatment (41%) Histology: pure ATC (71%), ATC+diferentiated component (29%)	Surgery (*n* = 48): TT (*n* = 33), subTT (*n* = 10), hemithyroidectomy (*n* = 5), removal/Dissection of LNs (*n* = 28) Resection status: R0 (12.50%), R1 (12.50%), R2 (58%), RX (17%) Targeted therapy (*n* = 3) ChT (*n* = 15): The most frequently used chemotherapeutics were Carboplatin, Cisplatin, Paclitaxel, and Doxorubicin in mono‐ or combined therapy CRT (concurrent) (*n* = 11) Induction ChT (*n* = 2) ChT after RT (*n* = 2) RT (98%): opposing field technique (32%), 3D‐conformal technique (18%), the combination of opposing field and 3D‐conformal technique (27%), IMRT (15%), VMAT (6%), unknown (2%) Median applied dose (Gy, range) 41.5 (4–66)	The median OS was 6 months, and 30% lived longer than one year Ten patients (16%) lived longer than 24 months, and three (5%) longer than 60 months (5 years) An extension of OS from 3.6 to 9.8 months with performing any surgery Better OS with R0 margin The median OS time was 13 months in patients who received a total radiation dose of 50 Gy or higher, vs 2.5 months in the comparison group (< 50 Gy). More prolonged survival (9.8 months) with MMT consisting of surgery, RT, and ChT, compared to the use of only one or two therapy modalities ChT or concurrent CRT had no significant effect on survival time, neither for patients with M0 nor for cases without metastases at the time of diagnosis. In patients with distant metastases (stage IVC), neither surgery nor ChT significantly affected OS.	Complete resection (R0), a total radiation dose of 50 Gy or higher, and the use of MMT were significant predictors for longer survival.	Radiation‐associated dysphagia and skin toxicity, a lower risk of dysphagia with a radiation dose of at least 50 Gy
Zhao, United States of America, 2023 [14]	Single‐Center retrospective cohort	57	G1: 53.1% F G2: 38.5% F G3: 41.7% F	G1: 61.5 ± 9.3 G2: 67.2 ± 11.3 G3: 68.6 ± 6.0	Stage: IVB (35%), IVC (65%)	NA	G1: neoadjuvant + surgery (*n* = 32) G2: only BRAF‐directed therapy (*n* = 13) G3: Upfront surgery (*n* = 12)	G1: 12‐month OS of 93.6% G2: 12‐month OS of 74.1% G3: 12‐month OS of 38.5%	G1: PFS of 84.4% [CI 71.8–96.7] G2: PFS of 50% [CI 48.7–99.5] G3: PFS of 15.4% [CI 0–35.0]	NA
Wu, United States of America, 2023 [15]	Single‐Center retrospective case series	97	F: 62%	70 (63–77)	IVA (19%), IVB (20%), IVC (55%)	Wild‐type BRAF 20.6%) Other coexisting tumor types (32.9%) Specimen with ATC tumor type only (12.3%)	ChT (55%) Definitive/adjuvant RT (65%) Surgery (55%)	The median OS of 6.5 months with a total of 81 deaths The improved median OS with receiving surgery vs. no surgery (10.3 vs. 4.1 months), ChT vs. no ChT (11.6 vs. 3.4 months), and definitive RT vs. no definitive RT (11.8 vs. 3.9 months) The longer median OS (6.7 months) in the 25 patients who received aggressive locoregional therapy (surgery and/or definitive/adjuvant RT) compared with the 28 patients who did not (2.8 months) among the 53 patients with distant metastases at diagnosis	NA	One patient experienced pneumonitis and then developed pancytopenia and hyponatremia Vemurafenib cessation because of fatigue, facial rash, and skin lesions.
Oliinyk, Germany, 2022 [16]	Retrospective cohort	123	F: 57.7% Not reported: 6.5%	71 (39–97)	Stage: IVC (100%)	NA	ChT record 76 (61.8%) ChT concurrent to RT (39.8%) ChT not concurrent to RT (8.9%) ChT without RT±surgery (9.8%) RT without ChT ± surgery (23.6%) No ChT and no RT ± surgery(14.6%) Sequence unknown (3.3%) RT record (76.4%): sufficient RT ≥ 30 Gy 80 (82.5%), RT dose unknown 2 (2.1%) Surgery (*n* = 39): TT (28.2%), subtotal or near‐TT (10.3%), lobectomy/hemithyroidectomy (5.1%), Debulking (12.8%), NA 17 (43.6%) Resection margins (*n* = 39): R0 (5.1%), R1 (43.6%), R2 (41.0%), NA (10.3) MMT (17.1%)	The OS rate of 27.1% and 7.9% at 6 and 12 months, respectively Improved OS with surgery (*p* < 0.001), administration of sufficient RT ≥ 30 Gy (*p* < 0.001), ChT (*p* < 0.001), and MMT (*p* = 0.014) Improved OS with TT	NA	NA
Al‐Jumayli, United States of America, 2022 [17]	Single‐Center retrospective cohort	35	F: 69%	70 (40–82)	IVA (37%), IVB (48%), IVC (14%)	The average size of tumor: 9 (3 –12) Distance metastasis: lung (8%), bone (2%), brain (2%)	Primary surgery (R0 resection 5%, R1 resection 5%, R2 resection 22%) followed by radiation therapy or CRT (34%) Taxol‐based CRT (37%) RT only (8%) ChT only (8%) Best supportive care (6%)	No statistically significant (p 0.6) difference in the OS between patients who underwent surgery followed CRT (3.2 months) and patients who received CRT therapy alone (3.6 months)	No statistically significant (p 0.6) difference in the PFS between patients who underwent surgery followed CRT and patients who received CRT therapy alone	NA
Park, South Korea, 2021 [18]	Single‐Center retrospective cohort	120	F: 67.5%	66.4 ± 11.3	IVA (5.8%), IVB (51.7%), IVC (42.5%)	Tumor size: 5.3 ± 2.4 LN metastases (77.5%) Distant metastases: lung (39.1%), bone (7.5%), other (83.3%) BRAF mutation: V600E (57.1%), wild‐type 5 (42.9%)	Surgery (64.1%): TT(54.1%), lobectomy (2.5%), partial/palliative resection (7.5%) RT (53.3%): the median cumulative dose of 45.00 Gy (range 10.00 ~ 68.40 Gy) Systemic therapy (24.1%): conventional ChT agents, including doxorubicin, paclitaxel, cisplatin, carboplatin, TKI therapy (15.8%): dabrafenib/trametinib, lenvatinib, vemurafenib	Median OS of 34.3 months for surgery and RT with TKI therapy, 21.4 months for surgery and RT with ChT, 8.2 months for surgery and RT, 5.3 months for radiation and ChT or TKI therapy, and 4.8 months for surgery and ChT	6‐month survival rates of 77.8, 76.2, 77.3, 32.1, and 37.5% for surgery and RT with TKI therapy, surgery and RT with ChT, surgery and RT, radiation and ChT or TKI therapy, and surgery and ChT, respectively	General weakness, weight loss, pneumothorax, and tracheoesophageal fistula
Kasemsiri, Thailand, 2021 [19]	Single‐Center retrospective cohort	121	F: 65.3%	≤ 40: 2.5% 41–50: 4.9% 51–60: 19.0% 61–70: 40.5% ≥ 70: 33.1%	IVA(30.6%), IVB (32.2%), IVC (23.9%), unknown (13.2%)	Tumor size (cm): < 5 6 (4.9%), ≥ 5 (72.7%), unknown (22.3%), extrathyroid invasion involved vital structures 14 (11.6) Distance metastasis: lung (22.6%), bone (4.0%), liver (1.6%)	Supportive treatment (30.6%) Palliative radiation (9.9%) Surgery alone (23.9%) CRT (7.4%) Surgery combined RT (10.7%) Surgery combined CRT (4.1%) Unknown (13.2%)	The one‐year OS rate of 3.5% (median OS time: 77 days) with a median follow‐up time of 74 days (range: 5–4,061 days) The median OS time of the interventional treatment (110 days) was almost twice as long and was significantly different from that of the palliative treatment group (58 days). The longest median OS time of 187 days and the longest survival rate of 20% in surgery with postoperative CRT	In stage IVA, the interventional treatment group (118 days) had significantly longer survival than the palliative treatment group (33 days, p ≤ 0.001); however, the median OS time of the interventional treatment group was not significantly longer than that of the palliative treatment group in stages IVB (intervention: 110 days); palliative: 63 days; p = 0.63;) and IVC intervention: 96 days; palliative: 64 days; *p* = 0.06;)	NA
Jonker, Australia, 2021 [20]	Single‐Center retrospective cohort	59	F: 59%	73 (39–99)	IVA (3.4%), IVB (47.5%), IVC (49.2%)	The median diameter of the primary tumor at initial diagnosis: 68 mm ATC percentage primary tumor: < 10% (5.1%), 10–50% (3.4%), > 50% (91.5%)	RT: < 40 Gy (20.3%), 40 to < 50 Gy (13.6%), 50 to< 60 Gy (13.6%), ≥ 60 Gy (20.3%), unknown dosage and/or fractions (5.1%) Surgery: thyroid surgery without LND (32.2%), thyroid surgery with LND (40.7%) Additional invasive interventions (30.5%) Systemic treatment: single treatment (42.4%), multiple treatments (22.0%), Molecular treatment(s): (11.9%) RAI: (10.2%) Limited treatment (42,3%), MMT (57.7%)	The median OS of 4.6 months, with a 1‐year OS of 23% The stage‐specific median OS benefit of MMT in stage IVB [6.9 (range 1.1–42) vs 3.0 (range 0.5–8.4) months; *p* = 0.003] and IVC ATC [5.6 (0.9–31.4) vs. 1.0 (0.1–5.3) months; *p* = 0.001] The similar median OS of stage IVC patients receiving MMT with or without TKIs and/or immunotherapy Superior PFS in stage IVB tumors with MMT[6.5 vs. 1.9 months; *p* = 0.005] as well as IVC in the MMT cohort [4.4 vs. 1.0 0.07–4.7) months; p, 0.001] MMT improved the median locoregional control and PFS more than LT	NA	The higher complication number in patients treated with MMT was caused by an increased number of grade 1 (*p* = 0.002), grade 2 (*p* < 0.001), and grade 3 (*p* = 0.02) complications
Houlihan, Ireland, 2021 [21]	Single‐Center retrospective cohort	7	F: 57.1%	58 (52–83)	T3aN0 (*n* = 1), T4bN1b (*n* = 3), T4bN0 (*n* = 3)	Median radiological tumor size: 50 mm (range 40–90 mm) Meadian pathological tumor size: 66 mm (range 43–110 mm) No distant metastasis Coexistent differentiated thyroid carcinoma (*n* = 2)	EBRT with concurrent doxorubicin (100%): 46.4 Gy in 29 bidaily fractions (*n* = 2), 60 Gy in 30 daily fractions (*n* = 2), 66 Gy in 30 fractions (*n* = 2), and 70 Gy in 35 fractions (*n* = 1) surgery followed by CRT (85.7%) neoadjuvant CRT followed by surgery (14.3%)	NA	The patient who received neoadjuvant CRT had a complete pathological response at the time of surgery. This patient remains alive and disease‐free at 9.8 months following surgical resection A combination of surgery and concurrent doxorubicin‐based CRT resulted in controlling local disease in 6/7 patients	Dermatitis, dysphagia, fatigue, anemia
Al‐Qurayshi, United States of America, 2021 [22]	Single‐Center retrospective cohort	1,870	F: 57.86%	69.0 ± 12.2	NA	Charlson/Deyo score: 0 (73.58%), 1 (20.21%), 2 (4.71%), ≥ 3 (1.50%) Metastasis at the time of diagnosis: 38.82%	Thyroidectomy only(16.33%), ChT only (4.11%), RT only (11.96%), Thyroidectomy + ChT (3.04%), Thyroidectomy + RT (10.32%), ChT and RT (25.13%), Thyroidectomy + ChT + RT(29.11%)	Compared to thyroidectomy alone, patients without metastasis who received thyroidectomy & RT, or ChT & RT, or thyroidectomy, ChT, & RT had better OS (P < 0.001 each). In patients with metastatic disease, any intervention or combination of interventions other than thyroidectomy alone improved survival (P < 0.05 each)	Decreased OS for patients older than 65 years with multiple comorbidities and presented with metastatic disease	NA
Gui, China, 2020 [23]	Retrospective cohort	1,404	F: 62.4%	70	NA	Tumor metastasis (56.1%), localized tumors (6%), regional involvement (35%), unstaged (2.9%) Tumor size: ≤ 2.0 cm (3.5%), 2.1–4.0 cm (10.7%), > 4 cm (52.3%), unknown (33.5%)	Surgery: lobectomy (17.4%), TT (26.4%), No cancer‐direct surgery/unknown surgical procedure (56.2%) RT (56.3%)	Significantly better OS and CSS for patients who underwent total or near TT than those who underwent lobectomy, and the worst survival patients with no cancer‐direct surgery or unknown surgical procedure Better survival in patients who received RT than did not receive RT	NA	NA
Fan, China, 2020 [24]	Single‐Center retrospective cohort	104	F: 52.9%	63.5 (28–87)	IVA (4.8%), IVB (73.1%), IVC (22.1%)	Tumor size cm: 6.5 (2.0–17.8) Coexistent differentiated thyroid carcinoma (37.5%) Extrathyroidal extension: Yes (94.2%), No (4.8%), Unknown (1.0%)	Surgical intervention (*n* = 55): 13 (12.5%) Resection status: R0 resection (n + 13), R1 resection (*n* = 22), R2 resection (*n* = 17) Concurrent ChT with RT (95.2%) Trimodal therapy with surgical resection followed by concurrent CRT (51%) Systemic therapy (*n* = 99): doxorubicin (73.7%), paclitaxel with or without pazopanib in a clinical trial setting (24.3%), other systemic agents (2.0%) IMRT (71.2%) EBRT: median dose 66.00 Gy (range 6.00–70.25 Gy) with a median of 33 fractions (range 3–40, BID (31.7%) or daily (68.3%)	The median OS for patients receiving ≥ 60 Gy was 10.6 months compared with 3.6 months among patients receiving < 60 Gy. Only the absence of metastases before RT (*p* = 0.009) and RT dose 60 (*p* = 0.004) were statistically significant factors for improving OS.	The 1‐year LPFS rate of 74.4% Only trimodal treatment (*p* = 0.017) and RT dose 60 Gy (*p* = 0.001) were associated with decreased risk of local progression.	The most commonly acute Grade 3 adverse events: dermatitis (20%), mucositis (13%), dysphagia (8%), and fatigue (7%) Subacute Grade 3 fatigue (*n* = 3) late Grade 3 fatigue (*n* = 4), grade 3 mucositis (*n* = 1)
Wachter, Germany, 2020 [25]	Single‐Center retrospective cohort	42	F: 45%	69 (33–89)	IVA (5%), IVB (52%), IVC (43%)	Distant metastases at initial diagnosis (43%) LN metastases at initial diagnosis (74%)	IVA/IVB: thyroidectomy + lymphadenectomy (52%), MMT including surgery ± ERBT ± ChT (48%), debulking/tracheostomy (14%), percutaneous endoscopic gastrostomy (*n* = 1) IVC: thyroidectomy + lymphadenectomy + metastasectomy + ERBT + ChT (16%), debulking/tracheostomy (22%), debulking/ChT + EBRT (28%), best supportive care± percutaneous endoscopic gastrostomy (33%) Resection status: IVA/B (*n* = 24); R0 (*n* = 6), R1 (*n* = 18) IVC (*n* = 18); R2 (*n* = 12), BSC (*n* = 6) EBRT radiation load: 45–70 Gy	IVA/B: After MMT, including surgery and EBRT plus/minus CTX, the median OS was significantly longer with a median of 25 (range 6–79) months than after surgery alone with 3 months (range 1 week to 38 months) (*p* = 0.04) in patients with a curative statement. The 3 patients with stage IVC carcinomas who underwent an MMT had a median OS of 6 (range 2–23) months, whereas stage IVC patients who had only debulking surgery and/or tracheostomies had a median OS of 7 (range 4–9) months. The median OS of the stage IVC patients after trimodal therapy were not significantly longer than after debulking procedures.	NA	NA
Maniakas, United States of America, 2020 [7]	Single‐Center retrospective cohort	479	F: 49%	65.0 (21.1–92.6)	IVA (11%), IVB (36%), IVC (53%)	BRAF V600E: 21.08%	Targeted therapy (33%): dabrafenib, trametinib, vemurafenib, cobimetinib, larotrectinib, everolimus, pazopanib, bevacizumab, lenvatinib, selpercatinib, lenalidomide, and cetuximab Immunotherapy (19%): pembrolizumab, atezolizumab, nivolumab, and ipilimumab Cytotoxic ChT (53%) Radiation‐locoregional (67%) Radiation‐other sites (13%) Surgery post neoadjuvant (5%): Neoadjuvant therapy included atezolizumab/cobimetinib (*n* = 2), docetaxel/doxorubic in (*n* = 1), and BRAFdirected therapies (combination BRAF plus MEK inhibitor): dabrafenib/trametinib (*n* = 13) or vemurafenib/cobimetinib (*n* = 7)	The median OS of 9.5 months 94% 1‐year survival with a median follow‐up of 1.21 years in patients undergoing surgery following neoadjuvant BRAF‐directed therapy (*n* = 20) Median OS for patients treated with targeted therapy, regardless of their grouping, was 1.31 years (15.7 months) compared with 0.63 years (7.6months) in patients not having received any targeted therapy	Sixteen of the 20 patients (80%) who underwent surgery following neoadjuvant BRAF‐directed therapy were alive at the last follow‐up, with a median follow‐up from diagnosis of 1.21 years	NA
Huang, China, 2019 [26]	Retrospective cohort	735	F: 62.6%	70	NA	Tumor size: ≤ 2.0 cm (3%), 2.1–4.0 cm (12.1%), 4.1–6.0 cm (21%), > 6.0 cm (40.4%), unknown (23.5%) Tumor extension: minimal extension (9.9%), extension to adjacent structures (59.7%), unknown (18.2%) LN involvement: N0 (35.6%), N1a(9.7%), N1b (31.3%), N1 (NOS) (8.3%), Nx (15.1%) Distant metastasis: no metastasis (46.8%), distant LN only (1.5%), distant metastasis ± distant LN (41.1%), Distant metastasis (NOS) (3%), unknown (7.6%)	No treatment (*n* = 159) ChT only (*n* = 27) Surgery only (*n* = 118) Surgery + chT (*n* = 19) RT only (*n* = 80) RT + ChT (*n* = 125) Surgery + RT (*n* = 56)	OS rates of 10.7% and 8.1% at two years and five years, respectively Surgery + RT could significantly improve OS compared with surgery alone (P = 0 004) The two‐year OS was 24.6%, 14.4%, and 2.4% in the TT group, less than in the TT group and no‐surgery group (P < 0 001). Compared with no LND, patients with LND had superior OS (P < 0 001)	Adding ChT to RT or surgery was more effective than RT alone or surgery alone Surgery + RT revealed significantly decreased HRs compared with other treatments Patients in the surgery + RT group showed a significant survival benefit than other groups. Patients with very locally advanced diseases (such as mediastinal tissue and prevertebral fascia extension, P = 0.380) or distant metastasis (P = 0.122) could not benefit from TT as well. Although LND could improve OS in patients who had thyroid surgery (P < 0 001), for their counterparts without thyroid surgery, the survival benefit disappeared.	NA
Corrigan, United States of America, 2019 [6]	Single‐Center retrospective cohort	28	F: 57.1%	70.9 (63.8 – 74.7)	IVA (7.1%), IVB (71.4%), IVC (17.9%), unknown (3.6%)	T stage: T2 (3.6%), T3 (3.6%), T4a (46.4%), T4b (42.9%), unknown (3.6%) N stage: N0 (32.1%), N1a (3.6%), N1b (14.3%), Nx or Unknown (50%) M stage: M0 (25%), M1 (17.9%), Mx or Unknown (57.1%) Lymphovascular invasion (50%) The extrathyroidal extension (82.1%)	Surgery (71.4%): lobectomy (*n* = 7), thyroidectomy (*n* = 12), LND (*n* = 9), metastasectomy (*n* = 1) RAI: after ATC diagnosis (*n* = 2), before ATC diagnosis (*n* = 2) EBRT: to any site (*n* = 21), to thyroid bed/neck (*n* = 19) Total radiation dose (cGy): < 4,000 (*n* = 11), ≥ 4,000 (*n* = 6), unknown (*n* = 4) Systemic therapy (50%) ChT agents: Doxorubicin, Carboplatin, Paclitaxel, Cyclophosphamide, Vinorelbine, Gemcitabine All treatments: Surgery + EBRT + ChT (*n* = 10), Surgery + EBRT (*n* = 5), EBRT + ChT (*n* = 3), EBRT only (*n* = 3), Surgery only (*n* = 5), ChT only (*n* = 1), No treatment (*n* = 1)	Median OS was 4 months, with a 1‐year survival rate of 17.9% for all patients The median OS after completing the first course of EBRT was 6 months, with a 1‐year survival rate of 23.8% as compared to a median OS of 1 month and 0.0% 1‐year survival rate in patients who did not receive EBRT. The median OS after the first surgical resection was 5.5 months, with a 1‐year survival rate of 25.0% as compared to a median OS of 1 month and 0.0% 1‐year survival rate in patients who did not undergo surgery. Patients receiving both surgery and EBRT had significantly better survival than those who received EBRT or surgery alone. Median OS after receiving ChT was 6 months, with a 1‐year survival rate of 21.4%. Receipt of EBRT and surgery were associated with improved OS.	NA	NA
Hamed, Egypt, 2018 [27]	Single‐Center retrospective cohort	54	F: 38.9%	≤ 60: 25.9 > 60: 74.1	NA	Tumor size: ≤ 5 cm (25.9%), > 5 cm (74.1) Extrathyroidal invasion (75.9%) Distant metastasis (29.6%)	Surgery: R0 (33.3), R1 (40.7%), other (26%) RT (66.7%) ChT (33.3%)	The 2‐year OS of 41.7% for stage IVA, 31.5% for stage IVB, and 7.4% for stage IVC (P = 0.04) The 2‐year OS rates of 59.3% for patients with negative margins, 30.1% for those with positive margins, and 0.0% in the group without thyroidectomy (P = 0.005) Better 2‐year OS (56%) in Surgery plus PORT compared to surgery alone (34.7%, P < 0.005)	NA	NA
Iwasaki, Japan, 2018 [28]	Single‐Center retrospective cohort	23	F: 69.6%	77.0 (42–89)	IVC (100%)	Metastasis: lung (82.6%), bone (13.0%), other (39.1%) Median size of tumor: 44.2 ± 17.8 mm (29.0–58.5)	TKI therapy: lenvatinib (100%) surgery before lenvatinib therapy (*n* = 8)	The median OS time of 166 days The greater median OS time for patients treated with surgery first (265 days) in comparison to patients treated with lenvatinib only (130 days)	The overall response rate of 17.4% and the disease control rate of 43.5% in levnatinib therapy	Treatment‐related AE for lenvatinib (100%): hypertension (91%), general fatigue and anorexia (65%), proteinuria (61%), and tumor‐skin fistulas (26%)
So, Australia, 2017 [29]	Single‐Center retrospective cohort	30	F: 56.7%	72	IVA (20%), IVB (47%), IVC (33%)	Median Tumor size (cm): 5.6 (3.7 – 8.8) The extent of disease at diagnosis: extrathyroidal extension (67%), LN involvement (57%) Metastasis: lung (*n* = 10), bone (*n* = 1), brain (*n* = 1), other (*n* = 3) Coexistent well‐differentiated thyroid carcinoma: papillary (*n* = 6), follicular (*n* = 1)	Surgery (*n* = 15): TT (*n* = 5), hemi‐thyroidectomy (*n* = 6), subtotal/Debulking (*n* = 2), palliative tracheostomy (*n* = 2) RT (*n* = 27): The median definitive dose of 60 Gy, delivered in 30 daily fractions and 60.29 Gy in 40 bi‐daily fractions Concomitant ChT (*n* = 6): doxorubicin (*n* = 3), cisplatin (*n* = 2), unknown (*n* = 1) Palliative ChT (*n* = 5)	The median OS of patients treated with definitive and palliative intent was 11.8 months (95% CI 9.2–14.4) and 3.6 months (95% CI 2.8–4.3), respectively. Five longer‐term survivors (greater than12 months; four patients with definitive/adjuvant intent, and only one patient with trimodal therapy)	All patients who had R1 resections and RT achieved loco‐regional control. Six of seven patients (85.7%), who had subtotal resections (R2) followed by RT maintained loco‐regional control. Eight of 15 (53.3%) patients who received RT but not surgery maintained loco‐regional control, five (33.3%) had progressive disease, and loco‐regional status in two patients was unknown. Seven of nine (77.8%) and 12 of 18 (66.7%) patients treated with definitive and palliative RT, respectively, achieved loco‐regional control.	One acute toxicity of grade 3 or above (oesophagitis) with concomitant CRT
Jacobsen, Norway, 2017 [30]	Single‐Center retrospective cohort	31	F: 58.1%	69 (26–87)	IVA (*n* = 2), IVB (*n* = 20), IVC (*n* = 9)	Tumor diameter: ≤ 5 cm (*n* = 14), > 5 cm (*n* = 17) N stage: NX (*n* = 6), N0 (*n* = 6), N1b (*n* = 19) distant metastasis: lungs only (*n* = 5), several sites (*n* = 2)	Surgery (*n* = 13): curative intent (100%) Tracheostomy (*n* = 6) RT (*n* = 31): been hyper fractionated accelerated RT (HART) at a total dose of 64 Gy in 4 weeks, fraction dose 1.6 Gy twice daily at least 6 h apart, Monday till Friday, together with weekly 20 mg doxorubicin	Median OS of 9 (2–106) months for 31 patients Best median OS, 19 months, in the 13 patients where both RT and surgery were possible as primary treatments	Fivefold risk of dying in patients not receiving surgery compared to patients who received surgery	Postoperative comorbidity (*n* = 6): postoperative bleeding (*n* = 2), postoperative infection (*n* = 5), postoperative bilateral recurrent laryngeal nerve paralysis (*n* = 1), chylous leak (*n* = 1)
Prasongsook, United States of America, 2017 [31]	Single‐Center retrospective cohort	48	F:40%	62 (37.0–88.0)	IVA (4%), IVB (57%), IVC (39%)	NA	Palliative intention (*n* = 18): Surgery (*n* = 5); R0 (*n* = 1); R1 (*n* = 1); R2 (*n* = 2), Combined CRT (*n* = 8), RT with or without ChT (*n* = 17) with median dose of 30 Gy, ChT agents: doxorubicin and docetaxel (*n* = 6), carboplatin and paclitaxel (*n* = 1), paclitaxel only (*n* = 1) Intensive MMT (N = 30): Surgery (*n* = 27); R0 (*n* = 8); R1 (*n* = 14); R2 (*n* = 4), Combined CRT (*n* = 30), RT with or without ChT (*n* = 30) with median dose of 66 Gy, ChT agents: doxorubicin and docetaxel (*n* = 19), carboplatin and paclitaxel (*n* = 5), doxorubicin only (*n* = 4), cisplatin only (*n* = 2), paclitaxel only (*n* = 0)	Palliative intention: Median OS in Stage IVA (NA), Stage IVB (4 months), and Stage IVC (3.7 months) 1 Year OS of 0% in both stages of IVB and IVC MMT: median OS in Stage IVA not reached, 22.4 months in Stage IVB, and 4.6 months in stage IVC 1‐Year OS and 2‐year OS of 100% in stage IVA 1‐Year OS and 2‐year OS of 0% in both stages of IVB and IVC	Palliative intention: CR (*n* = 2), partial response or stable disease (*n* = 2), progressive disease or not evaluable (*n* = 7)	60% hospitalization in the MMT group; 60% required at least temporary feeding tube placement (1 permanent and therapy‐associated), and 10% required at least temporary tracheostomy Other causes of hospitalization: septic shock, respiratory failure, fever, and venous thromboembolism
Baek, Korea, 2017 [32]	Multicenter retrospective cohort	329	F: 64.1%	< 70: 51.7% ≥ 70: 48.3%	IVA (17.3%), IVB (49.5%), IVC (31.3%), unknown (1.8%)	Tumor size: < 5 cm(45%), ≥ 5 cm (53.8%), unknown (1.2%) Distant metastatic sites: lung (70.9%), bone (14.6%), lung and bone (4.9%), multiple sites (> 3) (5.8%), others (3.8%) Disease extent: local (16.1%), regional (52.6%), systemic (31.3%)	No definite treatment (24.6%) RT/concurrent CRT (15.2%) Curative resection (28.6%) Curative resection and adjuvant RT/concurrent CRT (25.5%) Curative resection and adjuvant ChT (3%) ChT (3%) Dose of external irradiation: No (59.3%0, < 40 Gy (30.7%), ≥ 40 Gy (10%) TT (60.2%) Therapeutic neck dissection (57.8%)	The DSS rate of 28.5% for all the patient The best 1‐year survival rate of 50.2% in the patients who underwent curative resection and adjuvant RT or concurrent CRT A sufficient dose of external irradiation of 40 Gy and greater was associated with a better treatment outcome. Survival improvement in the patients who underwent aggressive management, including curative resection, RT, or concurrent CRT	Aggressive management was associated with a significant survival benefit in patients with stage IVB as well as stage IVA	NA
Wendler, Germany, 2016 [33]	Multicenter retrospective cohort	100	F: 52%	70.5 (38–92)	IVA (9%), IVB (32%), IVC (54%), not reported (5%)	The median diameter of the largest single tumor node (mm): 60 (12–160) LN metastases at initial diagnosis (55%) Distant metastases at initial diagnosis (54%) Differentiated tumor part: PTC (12%), FTC (5%)	Primary surgery (83%): thyroidectomy (43%), hemithyroidectomy (19%), two‐stage thyroidectomy (8%), completion of previous (6%) Thyroid surgery: tumor reduction (3%), explorative surgery (4%) No surgery (17%) Resection status: local R0 (14%), local R1 (27%), local R2 (52%), local Rx (6%) EBRT (81%): adjuvant (32%), local palliative (49%), distant metastases (11%) ChT (56%): Doxorubicin weekly (25%), paclitaxel weekly (9%), Paclitaxel+pemetrexed (8%), Doxorubicin+cisplatin (8%), Carboplatin+paclitaxel (14%), TKI (10%), other (10%) MMT: surgery, ChT, and EBRT (49%), Surgery and EBRT (19%), RT + ChT (4%), surgery+CTX (4%) Only surgery (11%) Only RT (9%) Best supportive care (4%)	Significantly improved OS with radical surgery (P = 0.012), EBRT (P = 0.008), any ChT (P = 0.003), and – most importantly – a multimodal therapeutic regimen of combined surgery, EBRT and ChT Statistically significant likelihood of longer OS with combined paclitaxel and pemetrexed in comparison to other chemotherapeutic regimens Significantly longer survival for EBRT ≥ 40 Gy compared with < 40 Gy in stage IVB patients (P = 0.017) and any kind of ChT vs. no ChT and EBRT ≥ 40 Gy vs. < 40 Gy in stage IVC patients (P < 0.0001 and P = 0.004)	NA	NA
Liu, China, 2016 [34]	Single‐Center retrospective cohort	50	F:48%	≤ 60 years: 52% > 60 years: 48%	IVA (30%), IVB (38%), IVC (32%)	Tumor size: ≤ 4 cm: 34%, > 4 cm: 66% T stage: T4a (38%), T4b (62%) LN metastasis (42%) Distant metastasis (32%)	Surgery: R0 resection (34%), R1 resection (42%), other (24%) RT (32%) ChT (16%) Treatment methods: surgery only (42%), surgery plus RT or ChT (34%), CRT (2%), ChT only (4%), tracheotomy, biopsy or other (18%)	The 1‐year and 2‐year OS rates (OS) of 48.0% and 26.0%, respectively, in all patients, with the 2‐year OS of 40.0% and 31.0% and 6.3% for stage IVA, IVB and IVC, respectively (P < 0.05) In stage IVA and IVB patients, combined surgery with RT improved OS, and the 2‐year OS were 50.0% and 35.7%, respectively, in the group with combined surgery with RT and the group with surgery with (P < 0.05) PORT improved local control rate in stage IVA and IVB patients (P < 0.05) no difference between the OS time of those treated with surgery and those with non‐surgical treatment In patients with distant metastasis (IVC)	NA	NA
Glaser, United States of America, 2016 [35]	Retrospective cohort	3,552	F: 59.7%	< 65: 31.6% ≥ 65: 68.4%	NA	Tumor size: < 6 cm (37.0%), ≥ 6 cm (32.0%), unknown (31.0%) Nodal classification: Clinically or pathologically positive (44.7%), negative/unknown (55.3%) Metastasis (41.6%) Surgical margins: negative (13.2%), positive/unknown (36.3%), no surgery performed (50.5%) Charlson–Deyo comorbidity score: 0 (54.6%), 1 (14.4%), ≥ 2 (4.1%), unknown (26.8%)	Surgery: TT (23.2%), other surgery (26.3%) EBRT (58.7%) ChT (41.6%): 57% single agent, 43% multiagent Both ChT and RT (33.1%): 75.0% concurrent, 9.9% sequential with ChT first, and 15.1% sequential with RT first	The unadjusted median OS of 3.5 months (IQR, 1.5–8.8 months) with 1‐year and 2‐year OS rates of 19% and 11%, respectively Significantly improved OS with any surgery; receipt of ChT; and receipt of RT > 36 Gy Association of negative surgical margins, TT, and RT to a dose of at least 59.4 Gy with long‐term survival	NA	NA
Paunovic, Serbia, 2015 [36]	Single‐Center retrospective cohort	150	F:64%	< 40: 1.3% 41–50: 6.1% 51–60: 19.3% 61–70: 54.0% > 70: 19.3%	NA	T1–T3 (3.6%) T4 (96.4%) Metastases in LNs (63.5%) Distant metastasis (32.9%) Pre‐existing well‐differentiated tumor (papillary) (57.6%) Lymphocyte infiltration (8.2%)	Surgery (*n* = 85): open biopsy (*n* = 12), tracheotomy (*n* = 2), tumour reduction (*n* = 27), lobectomy (*n* = 4), thyroidectomy and dissection (*n* = 40), multicentricity of tumour (*n* = 5) Preoperative RT (*n* = 2) PORT (*n* = 67) ChT (*n* = 2) RAI therapy (*n* = 8)	The mean survival time of 56 weeks Median OS time of 16 weeks, and 1 and 5‐year survival were 17% and 8% The longest survival in the radical surgery group: one year – 50%, five years – 27% Postoperative additional transcutaneous RT acts as a protective factor significantly associated with the survival rate	NA	NA
Goffredo, United States of America, 2015 [37]	Retrospective cohort	680	Surgery: 61.5% F No surgery: 57.1% F	Surgery: 68.6 ± 12.5 No surgery: 72.0 ± 11.6	Surgery: IVA (42.7%), IVB (32.2%), IVC (25.1%) No surgery: IVA in (14.2%), IVB (38.8%), and IVC (47%)	Surgery: tumor size of 59.2 ± 26.6 mm No surgery: with tumor size of 63.3 ± 27.8 mm	Surgical resection: Lobectomy (26%), TT(74%) ChT (*n* = 332): 43.4% of the surgery group, 38.3% of no surgery group RT (*n* = 327 in surgical group vs. *n* = 335 in no surgery group): none (36.7% vs. 39.7%), EBRT (44% vs. 52.8%), IMRT (13.5% vs. 7.5%), RAI (5.8% vs. none)	The median OS for patients who underwent thyroid resection was 9.7 months for those with stage IVA, 4.2 months for stage IVB, and 3.4 months for stage IVC (p\0.001). For patients who did not undergo surgery, the median OS for those staged as IVA was 3.0 months, IVB 3.4 months, and IVC 1.7 months, respectively (p\0.001). When stratified by stage, non‐surgical patients had shorter median OS than patients who underwent thyroidectomy for stage IVA (p\0.001) and IVC (*p* = 0.009) disease; survival was similar for patients with stage IVB (*p* = 0.074). The absolute differences in mean survival time gained by undergoing surgical resection were 23.4 months for patients with stage IVA ATC, 4.0 months for stage IVB, and 2.3 months for stage IVC disease. Stage IVA patients with negative margins experienced prolonged survival compared to patients who had R1 or R2 resections (*p* = 0.048). No differences in survival were observed for patients with disease staged IVB and IVC on the basis of their resection margin status Increasing time from diagnosis of ATC to surgery of up to 100 days was found to have no association with compromised survival for all disease stages	NA	NA
Aslan, Mexico, 2014 [38]	Retrospective cohort	29	F: 72.4	64.5 ± 13.2	IVA (10.3%), IVB 18(62.1%), IVC (27.6%)	T: T4a (10.3%),T4b (89.7%), N (%): N0 (37.9%), N1a (17.2%), N1b (44.8%), M Patients (%): M0 (72.4%), M1 (27.6%),	Surgery: TT (31%) with total resection (R0) in four patients, partial thyroidectomy 13.7% with R2 resection in all patients, incisional biopsy (10.3%) Concomitant RT with systemic therapy after surgery (*n* = 7) Surgery only (*n* = 2) Definitive treatment without surgery (*n* = 6) RT only (*n* = 2) No cancer treatment (*n* = 8)	The survival rates of 37.9%, 21%, and 13% at six months, one, and two years, respectively A better survival rate with radiation doses of higher than 33.1 Gy, complete resection (R0), and combined treatment (surgery with RT, with or without systemic therapy) (*p* = 0.006)	NA	NA
Brignardello, Italy, 2014 [39]	Single‐Center retrospective cohort	55	F: 61.8%	73.15	IVB (43.64%), IVC (56.36%)	Goiter 79.63%	Eligible for surgery (*n* = 41): early surgery alone (*n* = 5), early surgery + ChT (*n* = 12), early surgery + ChT + RT (*n* = 24), Not eligible for surgery (*n* = 14): neo‐adjuvant ChT + RT + surgery (*n* = 4), neo‐adjuvant ChT + RT (*n* = 2), neo‐adjuvant ChT only (*n* = 6), no treatment (*n* = 2)	The six‐month and one‐year survival rates of 49.09% and 19.70%, respectively The median OS of 5.55 months Better survival with maximal debulking than partial debulking without any difference between stage IV‐B and IV‐C patients The median OS time of 6.47 months and 1.48 months in eligible and non‐eligible patients, respectively. In patients eligible for early surgery, the “maximal debulking” was associated with longer survival, even in stage IV‐C patients	NA	Neutropenia: in most patients treated with cisplatin Symptomatic esophagitis in 20 out of 30 patients due to RT
Sugitani, Japan, 2014 [40]	Multicenter retrospective cohort	233	F: 68.2%	Group N: 71.0 ± 10.3 Group P: 69.2 ± 10.4 Group R: 67.7 ± 12.3 Group S: 68.5 ± 8.8	IVB (100%)	NA	23 patients (10%) underwent super‐radical surgery (group S); 49 patients (21%) received restricted radical surgery (group R); 72 patients (31%) had palliative surgery (group P); and 80 patients (34%) received no surgery (group N). No information regarding surgical procedures was available for the remaining 9 patients. Number of patients who underwent EBRT: No EBRT (%): group N (*n* = 35), group P (*n* = 23), group R (*n* = 11), group S (*n* = 11) < 40 Gy EBRT: group N (*n* = 9), group P (*n* = 8), group R (*n* = 5), group S (*n* = 3) > 40 Gy EBRT: group N (*n* = 34), group P (*n* = 38), group R (*n* = 33), group S (*n* = 9) The number of patients who received ChT: No ChT: group N (*n* = 37), group P (*n* = 40), group R (*n* = 19), group S (*n* = 14) Any ChT: group N (*n* = 41), group P (*n* = 30), group R (*n* = 30), group S (*n* = 9)	median OS (days): group N: 110, group P: 136, group R: 315, group S: 129 (*p* < .0001 (R vs N and P)) 6‐month CSS: group N: 28%, group P: 43%, group R: 68%, group S: 38% (p.0065 (S vs N and P)) 1‐year CSS: group N: 10%, group P: 15%, group R: 41%, group S: 33% (*p* = 0.94 (R vs S)) Number of patients who survived > 1 year: group N (*n* = 6), group P (*n* = 9), group R (*n* = 19), group S (*n* = 7) (*p* < .0001 (R and S vs N and P)) Number of patients who achieved complete remission: group N (*n* = 1), group P (*n* = 2), group R (*n* = 34), group S (*n* = 18) (*p* < .0001 (R and S vs N and P))	Aggressive surgery offers significantly better outcomes. than limited surgery for patients with TNM stage IVB ATC	Postoperative fistula (*n* = 4, group S): One of them eventually died of bleeding from the carotid artery. Other than those, one patient died as a consequence of a ChT‐induced adverse event.
Polistena, Italy, 2014 [41]	Retrospective cohort	79	F: 55.7%	72.6 ± 8.7	NA	G1: tumor less than 5 cm in main size (*n* = 45) G2: tumor larger than 5 cm (*n* = 34)	Surgery: G1: (*n* = 32): TT ± LND (90.6%) and 3 palliative lobectomy (9.4%) G2 (*n* = 12): TT (58.4%), lobectomy (16.6%), debulking surgery (25%). RT: G1: (*n* = 33), G2: (*n* = 15) RT without surgery: G1 (*n* = 9), G2 (*n* = 7) Tracheostomy: G1 (*n* = 21), G2 (*n* = 17) Endoprosthesis: G1 (*n* = 12), G2 (*n* = 8)	The mean survival was 5.35 (± 3.2) months with no significant difference in G1 vs. G2. Survival is significantly improved by surgery for G1 with 4.42 (P = 0.001) and G2 with 3.5 months (P = 0.0001) and by RT for G1 with 3.44 and G2 with 3.28 months (P = 0.047 and P = 0.0001, respectively) compared to no treatment at all which shows survival of 2.90 and 1.86 months, respectively, for G1 and G2. Considering the subgroup of patients who had undergone surgery, a significant difference in survival was observed comparing G1 vs. G2, respectively 6.93 and 5.75 months (P = 0.013). The MMT combining surgery with RT offered a significantly better outcome in tumors smaller than 5 cm compared to larger ones, respectively 7.73 and 6.20 months (P = 0.017).	NA	NA
Mohebati, United States of America, 2014 [42]	Single‐Center retrospective cohort	83	F: 50.6%	60 (28–89)	NA	Gross evidence of extrathyroid extension (*n* = 31) Clinical evidence of nodal disease (*n* = 23) Distant metastasis (*n* = 20)	Treatment types: no treatment (*n* = 7, 8%), surgery alone (*n* = 10, 12%), CRT (*n* = 4, 5%), RT alone (*n* = 3, 4%), surgery + PORT (*n* = 12, 14%), surgery + PORT + ChT (*n* = 38, 46%), surgery + ChT (*n* = 9, 11%) Treatment groups: surgery ± PORT ± ChT (*n* = 24, 29%), other (no surgery, or surgery alone) (*n* = 59, 71%) Resection type: R0/1 (*n* = 28, 34%), R2/X (*n* = 41, 49%), no surgery/treatment (*n* = 14, 17%)	The 1‐year DSS of 33% The median OS of the 7 patients who did not receive any therapy was 2 months, and no patient was alive at 1 year. 1‐year DSS: MMT: 42.4%, Single modality treatment: 6.0% (*p* < 0.001) 1‐year DSS: Surgery + RT: 8.3%, Surgery + CRT: 52.6% (*p* = 0.002) 1‐year DSS: R0/R1: 54.3% R2/Rx: 27.5% *p* = 0.021	multimodality therapy and an R0/R1 resection were statistically significant predictors of improved outcome patients with an R0/R1 resection were 2 times less likely to die compared with those with an R2/RX resection (p = .037), and patients treated with multimodality therapy were 3 times less likely to die (p= .013).	NA
Conzo, Italy, 2014 [43]	Single‐Center retrospective cohort	114	F: 59.7%	72.6 ± 8.7	NA	G1: the tumor's main size is less than 5 cm (*n* = 61) G2: the neoplasms larger than 5 cm (*n* = 53)	Surgery: G1: (*n* = 45). The related procedures included 40 TT ± LND (88.8%) and 5 palliative L (11.1%).G2: (*n* = 26) TT was performed in 17 patients (65.3%), L in 4 patients (15.3%) while 5 patients were suitable only for a debulking surgery (19.3%). RT: G1: (*n* = 44), G2: (*n* = 30) 23 cases (31.1%), respectively 12 of G1 (52.1%) and 11 of G2 (47.8%), were not considered for surgery but only for palliative treatment. In 15 of 74 cases (20.2%), ChT was associated. Tracheostomy: G1: (*n* = 28), G2: (*n* = 27) Endoprosthesis was positioned in 27 patients (23.7%), 16 cases (59.2%) of G1 and 11 cases (40.8%) of G2; in 19 cases (70.3%), further cervical surgery was not associated	The mean survival rate in all 114 patients was 5.35 ± 3.2 months, respectively 5.9 ± 3.09 months in the G1 and 3.52 ± 3.5 months in the G2 (*p* = 0.39). A significant difference in survival was observed when G1 vs G2 was compared (*p* = 0.013). The MMT combining surgery and RT resulted in better outcomes for smaller tumors, less than 5 cm, compared to larger lesions (*p* = 0.017). Despite the specific role of combined treatment, survival was significantly improved by surgery alone (G1: *p* = 0.001; G2: *p* = 0.0001) or by RT alone (respectively *p* = 0.047 and *p* = 0.0001)	NA	NA
Sun, China, 2013 [44]	Single‐Center retrospective cohort	60	F: 53.3%	58 (27–80)	NA	Common type (*n* = 47); incidental type (*n* = 5); anaplastic transformation at the neck LN(s) (*n* = 8); and no anaplastic transformation at a distant site Metastasis (*n* = 16): bilateral lung metastases in 9 patients, bilateral lung and bone in 1, bone in 3, and unilateral lung in 3	No treatment (*n* = 2), ChT alone (*n* = 6); RT alone (*n* = 6); surgical resection alone (*n* = 12); Only tracheostomy and/or biopsy (*n* = 5); MMTs (*n* = 29): 15 patients with surgery with PORT, 8 patients with surgery with postoperative CRT, and 6 patients with surgery with ChT	No significant difference in OS for the patients with the common type, the incidental type, or the type of anaplastic transformation at the neck was found; their median OS were 6, 7, and 8 months, respectively (P = 0.559). Among the 34 patients with upper airway obstruction, 5 patients who received tracheostomy had a worse prognosis (all died, 1 each at 2, 3, 4, 11, and 36 months after the initial diagnosis) than did those patients who did not receive tracheostomy (3‐ and 5‐year OS rates were 23.0% and 16.3%, respectively), although the difference was not significant (χ2 = 0.484, P = 0.487). The patients in the surgery plus PORT group had a better prognosis (P = 0.050) compared to the surgery group Among the 16 patients with distant metastasis, the median OS duration without ChT was shorter than with ChT, although the difference was not significant (2 months vs. 3 months, respectively; P = 0.859) Among the 44 patients without distant metastasis, the median OS duration without ChT was significantly longer than with ChT (11 months vs 7.5 months; P = 0.038).	NA	The distant metastasis rate was lower in the group that underwent surgery (7/41, 17.1%) than in the non‐surgical group (9/19, 47.4%)
Haymart, United States of America, 2013 [45]	Retrospective cohort	2,742	F: 61.9%	70 ± 12.29	IVA (37.5%), IVB (37.2%), IVC (25.3%)	NA	Surgery: TT (32.7%), lobectomy (17.5%), no surgery (47.8%), unknown (2.0%) RT: yes (58.2%), no (39.0%), unknown (2.8%) ChT: yes (38.8%), no (59.2%), unknown (2.0%)	The median OS with TT was 6.2 months vs 2.3 months without any surgery. Similarly, treatment with RT was associated with an almost 5‐month median OS vs close to 1.8 months without therapy. ChT improved median OS from 2.3 months to 5.9 months. The longest median OS in patients with Stage IVA was 11.2 months in patients who received surgery, radiation, and ChT Patients with Stage IVB had a 9.9‐month median OS when they received surgery with radiation and ChT and a 5.9‐month survival when they just received surgery and radiation. Patients with Stage IVC had a 4.9‐month median OS if they received surgery, radiation, and ChT and a 3.5‐month survival if they only received surgery and radiation	NA	NA
Brown, United States of America, 2013 [46]	Retrospective case series	38	F: 44.7%	64.5 (32–87)	NA	Metastasis (*n* = 18): (13 lung, 3 lung and bone, 1 lung and liver, and 1 lung, bone, liver, and spleen)	G1: Biopsy with or without tracheostomy (*n* = 22, 18 with distant metastasis, 4 with extrathyroidal extension of cancer lateral to carotid arteries): surgically unresectable, 4 of these patients received palliative radiation, 2 received palliative ChT, and the rest received supportive care. G2: Surgical resection (*n* = 16): 14 of which underwent postoperative EBRT varying from 50 to 70 Gy	G1, patients with distant metastasis: average survival time from date of biopsy 5.4 months (range, 1.5–8 months). G1, patients with extrathyroidal extension of cancer lateral to carotid arteries: average survival was 3.8 months (range, 3.0–5.8 months). all 4 patients had evidence of metastases by 2.5 months of follow‐up (3 lung, 1 lung, and bone). G2: no local recurrence, 6 with distant metastasis at an average follow‐up of 3.2 months, 1 died of an unrelated myocardial infarction at 3 months, 2 were lost to follow‐up, and 7 remain disease‐free with an average follow‐up of 4.8 years (range, 9 months to 8 years). Of those patients who underwent complete surgical resection followed by PORT, 7 of 14 (50%) are still alive, with a mean follow‐up of 4.8 years.	NA	G2: postoperative pharynx cutaneous fistulas in 2 of total laryngectomy patients, temporary hypocalcemia (*n* = 9), permanent hypocalcemia requiring long‐term calcium supplementation (*n* = 5), perioperative myocardial infarction (*n* = 1), postoperative pneumonia (*n* = 1)
Ito, Japan, 2012 [47]	Single‐Center retrospective cohort	40	F: 65%	74 (72.2 ± 8.6)	IVA (0.0), IVB (62.5%), IVB‐a (30.0%), IVB‐b (32.5%), IVC (37.5%)	Systemic metastases (*n* = 15): lung (*n* = 13), lung and bone (*n* = 1), lung and liver (*n* = 1)	Surgery: TT (*n* = 8), near‐TT (*n* = 1), subTT (*n* = 3), and palliative resection (*n* = 8) Concomitant neck dissection (*n* = 12) Tracheostomy (*n* = 17) ChT (*n* = 21): a combination of cisplatin and either 1 or 2 agents such as epirubicin, adriamycin, VP16 or 5FU (*n* = 16), Taxane monotherapy (*n* = 3), docetaxel (*n* = 2), paclitaxel (*n* = 1) External radiation (*n* = 29) Potentially curative surgery followed by EBRT and ChT (*n* = 6) RT and ChT (*n* = 11)	The median OS of 412 days (13.7 months) in patients with potentially curative surgery followed by radiation and ChT vs the MST of the 235 days (7.8 months) and 93 days (3.1 months) in patients treated with RT and ChT and RT alone, respectively No significant differences in survival times between the patients treated with 3 treatments and those with 2 treatments	NA	NA
Sugitani, Japan, 2012 [48]	Multicenter retrospective cohort	677	Common‐type ATC: F: 62%	Common‐type ATC: 68.7 ± 11.0 (28–100)	NA	Common‐type (*n* = 547); incidental type (*n* = 29); anaplastic transformation at the neck LN(s) (*n* = 95); anaplastic transformation at a distant site (*n* = 6)	Common‐type ATC: Stage IVA (*n* = 69); Surgery: None or palliative (*n* = 32), Radical (*n* = 36) External radiation: < 40 Gy (*n* = 28), > 40 Gy (*n* = 40) ChT: (*n* = 37) Stage IVB (*n* = 242); Surgery: None or palliative (*n* = 155), Radical (*n* = 80) External radiation: < 40 Gy (*n* = 110), > 40 Gy (*n* = 120) ChT: (*n* = 114) Stage IVC (*n* = 223); Surgery: None or palliative (*n* = 193), Radical (*n* = 27) External irradiation < 40 Gy (*n* = 144), > 40 Gy (*n* = 77) ChT: (*n* = 102)	Incidental type: MST = 395 days, and the 1‐year CSS rate = 57% (These values were significantly better than those for the other ATC types (*p* = 0.0001)). Survival for anaplastic transformation at the neck LN(s) (MST 175 days; 1‐year CSS 30%) was also better than that for the common type (*p* = 0.0082). Anaplastic change at a distant site had the worst prognosis (MST 48 days; 1‐year CSS 0%; *p* = 0.0001, compared to the common type). Long‐term survival (more than 1 year) (*n* = 84) in common‐type ATC: 52 (62%) underwent radical surgery, 64 (76%) had C40 Gy RT, and 58 (69%) received ChT, 29 (35%) underwent all three procedures	Common‐type ATC: Radical surgery compared to no or palliative surgery, C40 Gy of RT compared to no or\40 Gy RT, and any ChT compared with no ChT were associated with significantly better outcomes For stage IVA patients, radical surgery and C40 Gy of RT showed a significant association with better outcomes. Among patients with stage IVA common‐type ATC who underwent radical surgery, there was no significant benefit from adjuvant therapies, including RT and ChT. As for stage IVB patients, all three treatments (radical surgery, C40 Gy RT, ChT) were significantly associated with favorable outcomes. The three treatment methods were also associated with significant effects even in patients with stage IVC disease.	NA
Sosa, 2012 [49]	Multicenter randomized, controlled phase 2/3 trial	80	F: 53.8%	61.4 (28.0–83.0)	IV (*n* = 1), IVA (*n* = 1), IVB (*n* = 6), IVC (*n* = 72)	NA	Operative group (*n* = 44, 31 of whom with near‐total/TT): 30 on the CA4P arm, 14 on the control arm; 48% receiving radiation; 30% receiving ChT Non‐operative group: 25 on the CA4P arm, 11 on the control arm; 31% receiving radiation; 36% receiving ChT A total of 14 patients had a tracheostomy (*n* = 14): 10 were on the CA4P arm, and 4 were on the control arm. A tracheal stent (*n* = 1, on the CA4P arm), hemithyroidectomy, laryngectomy/pharyngectomy/thyroidectomy, and a clavicular resection (*n* = 3)	Median OS for patients who had the cancer‐related operation was 8.2 months in the CA4P arm vs 4.0 months in the control arm, resulting in a hazard ratio of 0.66 (P = .25) and a suggested associated reduction in risk of death of 35%. The 1‐year survival was 33.3% in the CA4P arm vs 7.7% in the control arm. The median OS for patients who had near‐total/TT was 10.0 months on the CA4P arm and 4.0 months on the control arm. Survival at 1 year was 35.0% on the CA4P arm and 10.0% on the control arm. Among patients who underwent less extensive operations (*n* = 13), the median OS was 4.9 months for those on the CA4P arm of the trial compared with 3.8 months for those on the control arm, with overlapping CIs. No associations between tracheostomy status and OS	NA	NA
Segerhammar, Sweden, 2012 [50]	Retrospective cohort	59	F: 71.2%	77 (49–95)	NA	Metastasis: local LN only (*n* = 11), distant only (lungs) (*n* = 11), local + distant (lungs/liver/brain/skeleton) (*n* = 22), no metastases (*n* = 14)	Neoadjuvant+ surgery (*n* = 36), discontinued neoadjuvant treatment (*n* = 19), no treatment (*n* = 4) surgery types (*n* = 36): TT (*n* = 19), lobectomy (*n* = 11), Lobectomy + contralateral resection (*n* = 6) Surgery/Margin (*n* = 36): radical/R0 (*n* = 8), intralesional/R1 (*n* = 23), tumor reduction/R2 (*n* = 5) Tracheostomy (*n* = 7)	Patients who underwent surgery had significantly longer survival than those who did not (median 5.4 and 0.8 months, respectively; P < 0.001) Patients Surviving > 12 Months (*n* = 6): Two patients died from distant metastases 12 and 22 months, respectively, after diagnosis. Three patients were alive with no evidence of disease at follow‐up (15, 32, and 160 months after diagnosis), and one patient was alive with a mediastinal metastasis and treated with stereotactic irradiation 58 months after diagnosis.	Follow‐up (*n* = 59): alive (all had been operated on) with no signs of ATC (*n* = 3) or with metastases (*n* = 1), dead from local tumor growth (*n* = 5) (of whom one had been operated on), dead from metastases (*n* = 50) (of whom 31 had been operated)	NA
Akaishi, Japan, 2011 [51]	Single‐Center retrospective cohort	100	F: 80	68	IVA (*n* = 11), IVB (*n* = 31), IVC (*n* = 58)	Tumor size (cm): ≤ 5 (*n* = 79), > 5 (*n* = 21)	Surgery (*n* = 70): TT (*n* = 35), subTT (*n* = 11), lobectomy (*n* = 1), complete resection (*n* = 24), extensive surgery (*n* = 9) RT (*n* = 78): 58 of them received a total dose of ‡ 40 Gy, PORT (*n* = 60), a combination of RT and ChT (*n* = 5), RT alone (*n* = 10) ChT (*n* = 28): ChT as adjuvant therapy after surgery (*n* = 25) MMT (*n* = 15): surgery, RT, and ChT	The median OS time of 3.9 months (range, 0.06–151.3) The overall 6‐month, 1‐year, and 2‐year survival rates of 40.4%, 21.3%, and 12.3%, respectively The median OS time was 33.5 months in stage IVA, 6.1 months in stage IVB, and 2.5 months in stage IVC. The 6‐month, 1‐year, and 2‐year survival rates were 67.8%, 53.2%, and 42.6%, respectively, after complete resection; vs 44.9%, 16.5%, and 3.9%, respectively, after debulking and 10.9%, 3.6%, and 0%, respectively, after no resection. The significantly higher survival rate after complete resection than after incomplete resection (debulking) or no resection (*p* < 0.0001) The higher survival rate in the group of patients irradiated with a dose of ≥ 40 Gy than the group irradiated with a dose < 40 Gy	In metastases‐free patients with complete tumor resection and metastases‐free patients with incomplete tumor resection, adjuvant RT was associated with significantly longer survival, but ChT was not. In patients with metastases, surgery and RT were associated with prolonged survival.	Pharyngoesophagitis, tracheitis, and bleeding into the airway in 20 patients following RT
Siironen, Finland, 2011 [52]	Single‐Center retrospective cohort	44	F: 73%	74	NA	Mean tumor size: 8 cm LN metastases (*n* = 15) Distant metastases (*n* = 29): mostly to the lungs	Surgery and CRT (*n* = 9), S and RT (*n* = 7), surgery and palliative CH + RT (*n* = 2), surgery (*n* = 13), CRT (*n* = 3), RT (*n* = 5), tracheostomy/stent (*n* = 2), none (*n* = 3) Thyroid surgery was classified as biopsy (*n* = 5), debulking (*n* = 15), lobectomy (*n* = 4), or TT (*n* = 11). Radicality of the surgery: none (*n* = 9), biopsy (*n* = 5), palliative (*n* = 21), or macroscopically radical (*n* = 9). RT classification higher dose (total dose > 40 Gy) or lower dose (< 40 Gy). At the lower dose (*n* = 8), a 2–3 Gy fraction was used. At the higher dose (*n* = 18), RT was given in 2‐Gy fractions daily or 1.3‐Gy fractions twice daily. ChT agents (*n* = 14): either doxorubicin‐based or paclitaxel. CRT (*n* = 14)	The longest median OS occurred in 9 patients undergoing MMT (surgery followed by CRT) and in patients undergoing radical surgery (11.6 months). Of 9 MMT patients, 4 underwent a radical operation, and their median OS was 3.2 years compared to 10.6 months for those whose operation was palliative. Elective operation (*p* = 0.008), RT (*p* = 0.002), ChT (*p* = 0.002), and CRT (*p* = 0.003) were also associated with more prolonged survival compared to survival without those treatments. Long‐time survivors (*n* = 3) (alive at last follow‐up or surviving 1 5 years): radical surgery + CRT or RT in all; one ATC survivor with a tumor infiltrating the recurrent nerve underwent only lobectomy, the rudimentary other lobe being left	NA	NA
Derbel, France, 2011 [53]	Single‐Center retrospective cohort	44	F: 59%	65 (44–80)	NA	Local (T4 N0 M0) (*n* = 12) Regional (T4 N1 M0) (*n* = 12) Distant (T4 N0 M1, T4 N1 M1) (*n* = 20)	TT (*n* = 28), near‐TT (*n* = 7), and debulking surgery (*n* = 9). Complete tumor resection (*n* = 8, 18.2%), microscopic residual disease (resection R1) (*n* = 9, 20.5%), and macroscopic residual disease (R2) (*n* = 25, 56.8%) RT to the thyroid area (*n* = 39): Hyperfractionated RT (*n* = 34), conventional radiation (*n* = 5) ChT (*n* = 38): a combination of doxorubicin and cisplatin (33), doxorubicin and carboplatin (*n* = 3), doxorubicin alone (*n* = 1), and paclitaxel (*n* = 1). Among the 35 patients who received the three‐phase combined treatment, 14 (41%) received the full six scheduled cycles of ChT. 8 patients received a second line of ChT after progression. 3 received paclitaxel, 3 received capecitabine, 1 received vinorelbine, and 1 had holoxan‐vepeside.	The median OS; in responders 28.4 months, and in progressive patients 5.1 months. (significantly different) with a median follow‐up of 7, 8 months (0,7, 160,6): 31 patients died, and the median OS was 8 months (6, 16,5). The median PFS was 6, 5 months (4,3, 12,5). 13 of the 44 treated patients are still alive, with a median OS of 8 months and a median PFS of 6.5 months. Median OS and PFS were significantly lower in the patients undergoing palliative surgery (debulking) than in those undergoing near‐total or TT (3.7 vs. 14.7 months respectively for OS, 3.7 vs. 10.6 months, respectively for PFS). No significant difference in median OS and PFS was seen between the patients with local stage ATC and those with loco‐regional or metastatic stage disease.	CR after treatment in14/44 patients (31.8%), partial response in 8 patients (18.2%), progressive disease in 22 patients (50%)	Surgery: unilateral vocal cord paralysis (*n* = 10), hemorrhage in the surgical site (*n* = 4), and hypocalcemia despite calcium supplement (*n* = 2) Toxicity: mainly digestive, with 65% grade 3 and 4 nausea and vomiting during treatment. Anemia and neutropenia were frequent among patients receiving doxorubicin and cisplatin. Grade 3 and 4 neutropenia was observed in 22.7% of the patients, whereas grade 3 or 4 thrombocytopenia was observed in 2.3%. The hematological toxicity was secondarily reduced by the systematic administration of G‐CSF and erythropoietin. Weight loss was noted in 65% of the patients in spite of nutritional support during RT. 1 patient had reversible renal toxicity.
Palestini, Italy, 2010 [54]	Single‐Center retrospective cohort	35	F: 65%	74 (57–88)	IVA/B (*n* = 10, 50%), IVC (*n* = 10, 50%)	Tumor maximum diameter: 2.5–12.0 (mean: 6,5)	Control group: submitted to partial tumor debulking or not operated at all (*n* = 15) Case group: resection in all patients (*n* = 20): Cervicotomy (*n* = 18) and cervicotomy plus partial sternotomy (*n* = 20) Deliberate resection of nervous/vascular structure (*n* = 6) Macroscopic residual disease (R2 resections) (*n* = 7) Tracheostomy (*n* = 1) Exeresis of an incidentally discovered parathyroid adenoma (*n* = 1) RT (*n* = 16): (36–40 Gy) combined with ChT (doxorubicin 20 mg/m2+ cis‐platinum 20 mg/m2) Only ChT (*n* = 1): paclitaxel 100 mg/m2	At the last follow‐up examination: 17 patients had died, 3 were alive 1, 6, and 80 months after the operation, the latter being free of disease. Survival of dead patients: 3 to 28 months (mean: 8 months); two patients lived > 1 yr and one lived > 2 yrs. In the control group, all the patients had died, survival from 1 to 13 months (mean: 4 months). Crude survival was better in our series than in the control group (*p* = 0.0112)	NA	Unilateral paralysis of RLN (*n* = 5, 25%): resulted from the deliberate en bloc resection of one RLN (*n* = 3) or vagus nerve (*n* = 1) or from the dissection of an RLN encased by the tumor (*n* = 1) Respiratory distress (*n* = 1) due to laryngeal edema and bilateral vocal folds hypomobility, which completely recovered after two‐days observation Seroma (*n* = 1), resolved after percutaneous aspiration
Swaak‐Kragten, Netherlands, 2009 [55]	Single‐Center retrospective cohort	75	F: 74.7%	68 (40–89)	IVA (9%), IVB (51%), IVC (40%)	40 patients with distant metastasis at presentation, mostly located in the lung	G1: patients treated before 1988 (*n* = 20; non‐protocol) G2: patients treated per protocol as of 1988 (*n* = 30) (hyper fractionated RT with low‐dose concomitant and adjuvant doxorubicin) G3: those patients treated after 1988 but outside the ATC protocol (*n* = 25; non‐protocol) Surgery: 63% of G2, 40% of G1, 36% of G3 Total radiation dose > 40 Gy: 87% in the G2 and only 11% and 17% in G1 and G3	Median OS 5.4 months in G2 vs. 2.8 and 1.9 months, respectively, in G1 and G3 (not significant) Long‐term survivors (more than 12 months): 6 patients 3 patients survived for more than 5 years; all had undergone R0/R1 surgical resection and CRT	CR: R0/R1 resection vs. R2 resection/debulking/biopsy: 63% vs. 12% (*p* < 0.001); 50% of the patients in G2, only 9% in the non‐protocol group (G1 + 3) (*p* < 0.001); CR rate of 89% in R0/R1 resection and radiation treatment according to protocol PLI (*n* = 15): 4 with pulmonary metastases at the start of this treatment (violation of protocol), In all four patients, the pulmonary metastasis progressed. Of the 11 M0 patients, nine remained free of lung metastases until the time of death or last follow‐up (82%). 1 out of 9 died of pulmonary fibrosis and bilateral pneumonia 9 months after treatment, being otherwise NED at the time of death. Of the four M0 patients in the protocol who did not receive PLI and lived more than 6 weeks after completion of treatment, only one remained free of lung metastases (25%).	Significantly worse toxicity in G2 No grade 4 toxicity in either group No interruption of treatment because of toxicity Almost half of the patients needed tube feeding because of severe radiation mucositis/esophagitis Organ toxicity in G2 vs G1 + 3: pharynx/esophagus 46% vs 11%, skin 7% vs 0%, mucous membranes 18% vs 0%, lung 13% in G2 and NR in G1 + 3
Yau, China, 2009 [56]	Single‐Center retrospective cohort	50	F: 68%	72 (36–104)	NA	Median tumor size: 6 cm (1–15 cm) Concomitant clinical detectable cervical LN metastases (*n* = 17) Distant metastases (*n* = 9)	Surgical resection: TT (*n* = 28), subTT (*n* = 3), lobectomy (*n* = 2), radiofrequency ablation of the tumor (*n* = 1), no surgery (*n* = 16) External RT to the neck (*n* = 23) Cytotoxic ChT (*n* = 18) either as part of a CRT regime or alone for metastatic disease	G1: long‐term survivors; died over a median of 6 years (3– 16 years) (*n* = 5) G2: died over a median of 3 months (4 days to 22 months) (*n* = 45) Surgical resection: 100% of G1 patients, 64% of G2 patients (P = 0.1) PORT: 80% of G1 vs. 42% of G2 (P = 0.1) ChT: 40% in G1 vs. 36% in G2 (P = 0.8)	No long‐term survivors had metastatic disease at presentation when compared with 25% metastatic disease detected in those who were not long‐term survivors. However, the difference was not statistically significant (P = 0.27). Moreover, all long‐term survivors had received surgical resection compared with 64% of those who were not (P = 0.1), and a higher proportion of long‐term survivors received PORT (80% vs. 42%, P = 0.1)	NA
Chen, United States of America, 2009 [57]	Retrospective cohort	261	F: 60.5%	70 (30–94)	NA	Tumor extension: confined within the capsule (*n* = 36), extension to adjacent structures (*n* = 110), further extension or metastasis (*n* = 95), the unknown extent of disease (*n* = 20) Tumor size: < 7 (*n* = 102), > 7 (*n* = 63), unknown (*n* = 96)	G1: EBRT alone (*n* = 37) G2: cancer‐directed surgery only (*n* = 68) G3: surgery plus EBRT (*n* = 129) G4: no treatment (*n* = 27)	Median OS (month) and 1‐year survival (%): G1: 3, 13.5% G2: 4, 24% G3: 6, 27.1% G4: 2, 11%	RT added to surgery resulted in improved survival for patients with disease extending into adjacent tissue. However, those patients who had disease confined to the thyroid capsule or had distant metastatic disease did not benefit from radiotherapy after surgery	NA
Brignardello, Italy, 2007 [58]	Single‐Center retrospective cohort	27	F: 66.7%	range 46–92	NA	The mean size of the tumor at the time of diagnosis: 6.0 ± 2.1 cm Tracheal infiltration (*n* = 11) esophageal infiltration (*n* = 8) Distant metastases (*n* = 15): most common in the lungs	Surgery: maximal debulking (*n* = 14), palliative resection (*n* = 6) adjuvant combined CRT (*n* = 15)	Significantly lower risk of death in patients treated by maximal debulking surgery than patients who underwent palliative resection or who were not operated at all	NA	NA
Kebebew, United States of America, 2005 [59]	Retrospective cohort	516	F: 66.9%	71.3	NA	Intrathyroidal ATC (*n* = 39) Extrathyroidal invasion and/or regional LN metastasis (*n* = 194) Distant metastasis (*n* = 222) Average tumor size: 6.4 cm (range, 1–15 cm)	Surgical resection (*n* = 253): TT (*n* = 12), subtotal or near TT (*n* = 37), lobectomy and/or isthmus‐ectomy (*n* = 23), removal of less than a lobe (*n* = 8), thyroidectomy, NOS (*n* = 2), surgery, NOS (*n* = 171) Radiation treatment: EBRT (*n* = 305), radioisotopeb (*n* = 12), unspecified (*n* = 9), none (*n* = 177), unknown (*n* = 13) EBRT sequence to surgical resection: before surgery (*n* = 4), after surgery (*n* = 147), before and after surgery (*n* = 4), no surgery (*n* = 143), unknown sequence (*n* = 7)	NA	The overall cause‐specific mortality of 69.4% at 6 months and 80.7% at 12 months combined surgical resection with EBRT treatment were independent prognostic factors for survival combined surgical resection with EBRT decreased the cause‐specific mortality rate significantly in patients with regional and distant disease but not in patients with only intrathyroidal ATC	NA
De Crevoisier, United States of America, 2004 [60]	Retrospective cohort	30	F: 60%	59 (40–79)	TNM stages: T4N0M0 (*n* = 7), T4N1M0 (*n* = 13), T4N0M1 (*n* = 2), and T4N1M1 (*n* = 4)	Concomitant follicular or papillary carcinoma (*n* = 14) LN involvement (*n* = 8) Initial distant metastases (*n* = 6)	Initial surgery in patients without distant metastasis (*n* = 17): TT (*n* = 11), partial thyroidectomy (*n* = 6), LND (*n* = 10) 2) Surgery in patients with initial distant metastasis: TT (*n* = 1), partial thyroidectomy (*n* = 2), LND (*n* = 1), complete resection of the neck tumor (*n* = 1) ChT and RT without initial surgery in patients without initial distant metastasis (*n* = 7) EBRT: after two cycles of ChT (*n* = 27), after one cycle of ChT (*n* = 3) The total dose of RT: 40 Gy (*n* = 29), 30 Gy (*n* = 1)	OS rates at 1 year and 3 years were 46% and 27%, respectively. The median OS was 10 months. Disease‐free survival rates at 1 year and 3 years were 38%and 24%, respectively. Macroscopic complete tumor resection was a significant factor in OS.	Complete local response at the end of the treatment (*n* = 19) complete remission in 8 of 17 patients with initial surgery at the end of the study, with a median follow‐up of 35 months (range, 5–78 months) Associated complete surgery with better survival in patients without distant metastases (*p* = 0.02)	Pharyngoesophagitis: grade 3 (23%), grade 4 (10%) Neutropenia: grade 3 (3%), grade 4 (70%) Thrombocytopenia: grade 3 (3%), grade 4 (10%) Anemia: grade 3 and 4 (27%) Reversible renal toxicity (*n* = 1) Grade 1 neurologic toxicity (*n* = 7)
Kihara, Japan, 2004 [61]	Single‐Center retrospective cohort	19	F: 63%	73.4 (45–87)	NA	Mean tumor size: 5.7 cm Extrathyroidal invasion (84%) Distant metastasis (47%): lung (*n* = 9), bone (*n* = 1)	Surgery (*n* = 10): complete resection (*n* = 4) RT (*n* = 13): dose of 45 Gy (*n* = 9), dose of < 45 Gy (*n* = 4) ChT (*n* = 12): doxorubicin alone or in different combinations (*n* = 9), paclitaxel (*n* = 1) Adjuvant therapy after surgery (*n* = 7)	The median OS of 9.4 months, with a range of 0.6–76.3 months, and the 1‐, 2‐, and 5‐year survival rates of 21%, 11%, and 5%, respectively A significantly better outcome in the 10 patients who underwent surgery than the 9 patients who did not (P = 0.0011) Significantly better survival after complete resection than that after incomplete resection or no surgery	Incomplete resection was the most important and independent factor for predicting death from ATC	NA
Pierie, United States of America, 2002 [62]	Single‐Center retrospective cohort	67	F: 67.2%	73 (40–92)	NA	Median tumor size: 7 cm (2–20 cm) Distant metastases (49%): mostly in the lung (28 of 33; 85%) but also in the brain, liver, and bone	Resection (*n* = 67): complete resection (*n* = 12), incomplete resection (*n* = 32), no resection (*n* = 23) EBRT (*n* = 56): with a dose of more than 45 Gy (*n* = 27) and less than 45 Gy (*n* = 29) ChT (*n* = 21), tracheostomy (*n* = 17), gastrostomy (*n* = 7)	6‐month and 1‐ and 3‐year survival rates: Complete resection: 92%, 92%, and 83% Incomplete resection: 53%, 35%, and 0% No resection: 22%, 4%, and 0% EBRT < 45 Gy: 31%, 17%, and 10% EBRT > 45 Gy: 66%, 54%, and 18% No change in survival following ChT, tracheostomy, and gastrostomy	NA	Complications in 12 (21%) of the 63 patients who were treated by surgery, EBRT, or both Unilateral recurrent nerve palsy in 2 (8%) of 24 patients who had no voice changes at presentation and underwent surgery with or without RT Radiation‐induced swallowing problems and feeding tubes in 7 (13%) of 56 patients for a median of 4 weeks (range, 1–10 weeks)
McIver, United States of America, 2001 [63]	Single‐Center retrospective cohort	134	F: 60%	67	NA	Metastases at diagnosis (46%): pulmonary or mediastinal metastases, skeletal metastases, brain metastases Distant metastases (68%)	Surgery (*n* = 83): debulking procedures (*n* = 48), curative procedure (*n* = 35) TT (13%), near‐TT (29%), lobectomy (46%), complex operation with curative intent by radical or modified‐radical neck dissection (12%) Residual status: complete resection (30%), minimal residual disease (26%), gross residual (44%) EBRT: gross residual disease (*n* = 41), total or near‐total resection (*n* = 38), after open biopsy (*n* = 29), local recurrences (*n* = 5), metastatic disease (*n* = 4) ChT: alone (*n* = 4), postoperative adjunctive therapy (*n* = 12) Palliative therapy (*n* = 5)	The median OS time of 3 months, with 32 patients (23%) surviving less then one month from the time of diagnosis Both operation and RT improved survival over palliative treatment alone, with patients who received only palliative care having a median OS of 3 weeks compared with 2.3 months for patients treated with RT (P < .005) and 3.5 months for patients treated surgically Neither the extent of operation nor the achieved completeness of resection had a significant impact on survival, with a median OS of 2.3 months for patients with gross residual disease after operation and 4 months for patients with complete tumor resection (P = .3).	NA	NA
Besic, Slovenia, 2001 [64]	Single‐Center retrospective cohort	79	F: 73%	40–86	NA	Tumor extension: intrathyroidal G1 (*n* = 8), G2 (*n* = 3); extrathyroidal G1 (*n* = 18), G2 (*n* = 50)	G1: primary surgery group (*n* = 26) G2: primary ChT and/or RT group (*n* = 53), including the 12 patients in whom surgery was performed after ChT and/or RT	No statistical difference in the survival between the primary surgery group and the primary ChT and/or RT group The longest survival (median 14.5 months) was obtained in the patients in whom primary ChT and/or RT reduced the tumor and thereby made its radical removal	NA	NA
Busnardo, Italy, 2000 [65]	Single‐Center retrospective cohort	39	F: 71.8%	69 (39–88)	NA	Local disease (*n* = 26) Distant metastases (*n* = 22): mainly to the lung at diagnosis or quickly developed during the observation period in all the others except one	G1 (*n* = 16): patients treated with surgery, plus RT, all but two, and ChT, all but one, G1A: consisted of 9 patients who were treated with the MMT scheme, G1B: 7 patients who had previously been treated by surgery and RT in other hospitals and treated by ChT in G2 (*n* = 9): patients treated with ChT alone G3 (*n* = 14): patients treated with RT alone (*n* = 4) or no treatment at all (*n* = 10)	Four CR were seen in patients from Group 1, and 1 from Group 2. One patient without distant metastases at diagnosis is alive and free of disease 6 months after TT and adjuvant ChT and 12 months after diagnosis. Three had long‐term survival (14, 24, 27 months) with a disease‐free interval of 6‐8‐10 months. The patient from Group 2, who was treated for a second time by TT, is alive without disease after 60 months. The median OS rate was 11 months for Group 1, 5.7 months for Group 2, and 4 months for Group 3. In some patients, MMT (TT, RT, and ChT) is associated with increased survival. Nine out of 16 patients who underwent surgery and complementary treatment had no local progression. In all patients except one, distant metastases developed, mainly in the lung, during or after post‐surgical CHT.	NA	NA
Lu, Taiwan, 1998 [66]	Retrospective cohort	46	F: 57.4%	63.3 ± 1.45 (46–84)	NA	Advanced ATC without distant metastases outside the neck area (G1: 43.5%) and with distant metastasis (G2: 56.5%) mainly to the lung Tumor size in the non‐surgical and surgical subgroups: 8.2 vs. 9.8 cm in G1, 7.34 vs. 8.43 cm in G2	Surgical and non‐surgical subgroups in both G1 and G2	G1: mean survival of 12.8 months vs. 8.6 months in the surgical and non‐surgical subgroups (*p* = 0.46) G2: mean survival of 3.5 months vs. 2.8 months in the surgical and non‐surgical subgroups (*p* = 0.72)	NA	NA

**Abbreviations:** AE, Adverse events; ATC, Anaplastic thyroid carcinoma; ChT, Chemotherapy; CA4P, Combretastatin A‐4 phosphate; CR, Complete response; CRT, Chemoradiation therapy; CSS, Cancer‐specific survival; DSS, Disease‐specific survival; EBRT, External beam radiation therapy; FTC, follicular thyroid carcinoma; G, Group; IMRT, intensity‐modulated radiation therapy; JAK, Janus kinase; LN, Lymph node; LND, Lymph node dissection; LPFS, Locoregional progression‐free survival; MMT, Multimodal treatment; MST, median survival time; NA, Not applicable; NR, Not relevant; OS, Overall survival; PFS, Progression‐free survival; PLI, Prophylactic lung irradiation; PORT, Postoperative radiation therapy; PTC, Papillary thyroid carcinoma; RAI, Radioactive iodine; RLN, Recurrent laryngeal nerve; RT, Radiation therapy; SD, Standard deviation; TKI, Tyrosine kinase inhibitor; TT, total thyroidectomy; Vs, versus

### 2.1 Search Strategy

A systematic search was performed in PubMed, Web of Science, and Scopus to identify potentially eligible studies (HN conducted the systematic search under the supervision of the corresponding authors [AJ and MS], and NH verified and double‐checked it). A complete list of search terms, including keywords and MeSH terms, is available in supplementary documents (Table S3).

### 2.2 Eligibility Criteria and Study Selection

In this systematic review, retrospective cohort studies with an available English full text published from the initiation until June 1st, 2023, were eligible for inclusion. The eligible source populations were individuals of any age who were suffering from anaplastic thyroid carcinoma, receiving surgery with or without other treatment types. Additionally, non‐English abstracts, guidelines, preclinical evaluations, experimental studies, reviews, case reports, case series, conference abstracts, and editorial letters were excluded.

### 2.3 Data Extraction

The data extraction processes for all eligible studies were developed separately by two independent investigators (NH and AH) as follows: (I) study characteristics (author, year, design, sample size, grade of thyroid cancer, treatment), (II) patients' characteristics (mean age and gender distribution), and (III) outcome (treatment efficacy, safety, and adverse events, follow‐up). The corresponding authors (AJ and MS) meticulously reviewed any discrepancies and disagreements to produce the final table containing the extracted data.

### 2.4 Risk of Bias Assessment

Two investigators (AH and YG) independently evaluated the risk of bias and methodological quality of the included studies. For these assessments, we utilized the National Institute of Health (NIH) Quality Assessment Tool for Cohort and Cross‐Sectional Studies [12] (Table S4).

## 3 Results

### 3.1 Search Results

**Figure 1:**
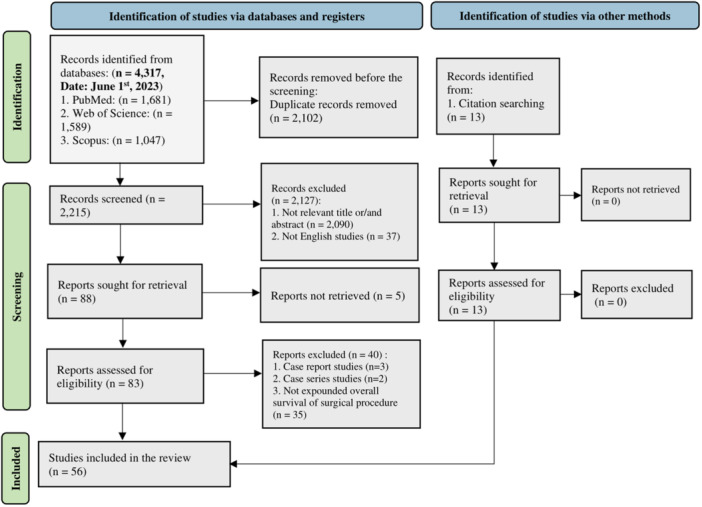
PRISMA 2020 flow diagram for systematic reviews, which included searches of databases and citation searches.

A total of 4,317 articles were detected in the search from initiation up to June 1st, 2023. A total of 2,102 duplicates were removed. In the first and second phases of the screening, 2,215 studies were reviewed by two independent reviewers (NH and AH) using the titles and abstracts to select the appropriate studies. Disagreements between the reviewers were resolved with discussion or the consensus of the third reviewer or the corresponding authors (AJ and MS). In this step, we excluded 2,127 articles. In the last screening phase, full texts of 83 articles were evaluated based on the predefined inclusion criteria. Finally, a total of 43 studies were included for data extraction. Also, 13 eligible studies not found in our initial search were included after searching the references of the included papers. Figure 1 demonstrates the study's PRISMA flowchart. Furthermore, all included studies achieved a “Good” score regarding the bias assessment results.

### 3.2 Study Characteristics

Finally, 56 studies met our study inclusion criteria and evaluated the impact of surgery alone as well as in comparison with other treatment methods on the survival of 16,246 patients suffering from ATC. A comprehensive detail of demographics, characteristics, and overall OS of included studies is illustrated in Table 1.

### 3.3 Surgery in the Management of ATC

Several investigations have demonstrated that curative surgery, as a positive prognostic factor in ATC management, was associated with prolonged OS [6, 13, 14, 15, 16, 17, 18, 19, 20, 21, 22, 23, 24, 25, 26, 27, 28, 29, 30, 31, 32, 33]. The patients who received surgery as the primary treatment experienced extended survival outcomes compared to other patients [6, 24, 25, 34]. According to the type of surgery, total tumor resection and complete microscopically negative margin (R0 margin) provided more favorable results than other surgery types [14, 15, 16, 17, 19, 20, 22, 25, 28, 29, 34, 35, 36, 37, 38, 39, 40, 41, 42, 43, 44, 45, 46, 47, 48]. However, in the cases that were not eligible for complete tumor resection and R0 margin, sub‐total and near‐total thyroidectomy were associated with superior results than other types of surgery, such as palliative surgery, or not performing surgery [7, 14, 16, 22, 35, 40, 45].

Regarding staging, eligible patients for aggressive surgical resections in all stages of ATC demonstrated prolonged OS than the patients who received not curative debulking or palliative surgery [7, 22, 23, 26, 27, 29, 49, 50, 51]. However, patients with distant metastasis and advanced stages of ATC showed higher recurrence rates and significantly lower OS after surgery in comparison with the subjects without distant metastasis and lower stages of ATC [14, 17, 34]. In detail, a higher number of patients with localized ATC, such as the IVA stage, received curative surgical resection of the tumor and depicted a significantly longer mean survival time compared to the patients with advanced ATC [17, 26, 50, 52, 53]. Nonetheless, in some studies, negative margin status did not have a significant effect on the survival outcomes of patients with IVC stage [26].

### 3.4 Multimodalities Treatments

Several investigations have illustrated that multimodality treatment, including surgery in combination with ChT, RT, and external beam radiation therapy (EBRT), significantly improves the OS of ATC patients [6, 7, 15, 18, 22, 24, 25, 27, 28, 29, 31, 32, 38, 40, 42, 43, 44, 47, 49, 51, 54, 55, 56, 57, 58, 59, 60, 61, 62]. Furthermore, in a number of studies, the combination of curative surgery with Chemoradiation therapy (CRT), especially RT therapy, has been correlated with extended OS and less locoregional recurrence compared to performing surgery alone or CRT alone [6, 7, 15, 18, 24, 25, 27, 28, 38, 45, 47, 49, 55, 56, 57, 59, 63]. However, in some studies, no significant difference in the survival outcomes was reported between multimodality management and surgery alone [37, 54]. According to the type of multimodality approach, high dose‐RT or EBRT was more effective than palliative dose in prolonging the OS survival of ATC cases, regardless of staging [6, 14, 25, 27, 29, 37, 47, 55].

Regarding staging, subjects with IVA/B stage of ATC who were treated with multimodality treatment (combination of surgery with ChT, RT, or both) showed a significant improvement in survival outcomes compared to the patients in the same stage who received surgery only or palliative therapy [23, 27, 38, 47, 58, 64]. Moreover, combination therapy has been associated with better local control of the disease in IVA/B patients compared to surgery alone [38]. Howbeit, in a study conducted by Kasemsiri et al., the median survival time did not demonstrate a significant difference between palliative and interventional groups in IVB ATC patients [65]. In some investigations, multimodality approach was associated with prolonged OS in cases suffering from distant metastases and IVC stage of the disease [22, 27, 58, 60, 64]; however, some other studies did not demonstrate a significant improvement in the OS with aggressive combination therapy [47, 53, 63, 65, 66], highlighting the fact that other factors can affect the treatment response in advanced stages of ATC.

## 4 Discussion

This systematic review comprehensively assessed the efficacy of surgery in the setting of unimodal therapy or combination with other treatment approaches on the survival outcomes of 16,246 patients affected by ATC in 56 cohort studies. In summary, the majority of previous studies endorsed the patients eligible for surgery, particularly when maximal resection along with R0 margin can be achieved, experienced more extended OS than patients who couldn't undergo surgical procedures. Further, most evidence illustrates that surgery combined with other treatment methods is more efficacious than surgery alone, especially in patients without distant metastases. However, the results may vary depending on the severity and stage of the disease.

ATC is the most uncommon but aggressive form of thyroid cancer, responsible for approximately 14%–39% of thyroid carcinoma‐related deaths [67]. Various RT, surgical intervention, and ChT types can be used in conjunction or separately to enable the best systemic and local disease control. In terms of treating patients, the mode of utilizing combination or divided treatment options should be adjusted based on the patient's disease stage and conditions, such as tumor size and systemic or local metastases. Based on the last update of the American Thyroid Association Guidelines for Management of Patients with Anaplastic Thyroid Cancer, most stage IVA/IVB cases with resectable tumors meet a favorable prognosis, specifically if multimodal therapy (surgery, RT, and systemic therapy) is employed [10]. Some stage IVB patients with unresectable cancer may benefit from the aggressive therapy method. It is also recommended that surgery be kept in mind if maximal resection is possible for patients with stage IVC disease. Recently, molecular targeted therapy and immunotherapy drug agents have been found to be particularly effective for treating challenging diseases and achieving optimal results [68, 69]. Therefore, molecular targeted therapies, including combinations of BRAF inhibitors with mitogen‐activated protein kinase kinase (MEK) inhibitors, as well as immunotherapies like Janus kinase (JAK) inhibitors and Tyrosine kinase inhibitors (TKIs), have shown efficacy in treating anaplastic thyroid carcinoma (ATC). This is particularly true for patients carrying the BRAF^V600E^ mutation, given the high mutation rate observed in ATC [5, 21, 70]. Nevertheless, a definitive treatment approach has yet to be developed, and current therapeutic options mainly focus on extending OS and enhancing the quality of life along with minimizing adverse events.

Thyroid surgery approaches, including total thyroidectomy, subtotal or near‐total thyroidectomy, and debulking surgery utilized in a multidisciplinary board based on the tumor size and/or invasion into the surrounding structures, as well as the extent of metastatic disease, that is, T‐/N‐/M‐stage [22]. Most of the guidelines have proposed total thyroidectomy with maximal resection if conceivable [10, 71, 72]. We have detected that performing surgery is associated with superior survival outcomes as several large‐scale investigations with a high percentage of IVC patients showed improved OS after surgery [14, 20, 27, 37, 45, 60]. Previous studies have indicated that achieving a negative margin after resection is linked to improved survival outcomes compared to lobectomy or debulking surgery [14, 22, 37, 42, 48, 52]. Additionally, some studies have evidence that palliative debulking procedures with or without ERBT and/or ChT resulted in more prolonged survival than just best supportive care alone at advanced stages of the disease [27, 53]. Nonetheless, patients at advanced stages could not benefit the most from this treatment method, and debulking surgery could postpone EBRT or systemic ChT because of wound complications [10, 65]. Moreover, ATC's aggressive nature provides a basis for super radical surgery such as laryngectomy or trachea resection, which has demonstrated beneficial results in IVB patients in the Sugitani et al. study [41]; however, the study by Goffredo et al. did not demonstrate survival benefits in IVB or IVC patients [26]. Additionally, some studies have demonstrated that cancer patients with tumors smaller than 5 cm benefit more from surgical procedures [33, 62]. These findings highlight the significance of total resection and achieving an R0 margin for better outcomes. Nevertheless, most patients with ATC present with extensive disease or metastasis at initial diagnosis, rendering them inoperable or suitable only for palliative care [73]. Therefore, patients with unresectable tumors or metastasis should receive aggressive therapy, such as cytotoxic chemotherapy, as initial therapy [10].

In addition to the standalone use of surgery, incorporating surgery with other treatment modalities such as RT, ChT, and neoadjuvant therapy proves beneficial for most patients by optimizing OS and progression‐free survival [6, 20, 23, 59, 63, 74]. Although recruiting the combination of surgery with CRT is already accepted as the standard of care in stage IVA and resectable stage IVB [71, 72, 75], multimodal therapy is also being investigated in patients with stage IVC and unresectable IVB and in some studies has been associated with positive effects on survival sequelae [22, 26, 76]. On the other hand, in some studies, no significant difference was observed between employing surgery alone and combination treatment [19, 44]. Indeed, multimodality management, including surgery, if applicable, plus RT and ChT, is more likely efficacious in the primary stages of the disease [29, 47, 52, 63]. Overall, these findings support that multimodal treatment can provide desirable outcomes, most notably when surgery is feasible.

It is also imperative to note that the timing and sequencing of surgery, as well as CRT, plays a crucial role in the management of ATC [10]. The findings of previous investigations exhibited the advantageous role of primary surgery for obtaining more favorable results, which was in line with the guidelines' recommendation on primary surgery with or without following adjuvant therapies if the tumor is resectable [10, 71]. Therewith, more studies evidence the effectiveness of postoperative CRT in achieving satisfactory outcomes [18, 50, 65]. One of the most recent analyses of the SEER database found that CRT was associated with improved OS than EBRT alone [77]. Notably, R0/R1 margin surgery combined with RT at all stages demonstrated superior outcomes in both OS and locoregional control [6, 45, 53, 55, 60]. In the case of unresectable primary tumors, neoadjuvant therapy may be a potential option for selected patients, which can be followed by upfront surgery [10, 72]. The dose of EBRT is crucial to ensure the optimal survival benefit to patients. Evidence suggests that the total dose delivered is predictive of both survival and local control in most studies. Based on the treatment goal, the radiation doses vary between 20 and 75 Gy [75]. An optimal control was generally achieved with doses ranging from 45 to 60 Gy, regardless of the fractionation type, as indicated by various studies [29, 59, 74, 78]. While in some studies, a cumulative dosage of ≥ 40 Gy was used for therapeutic purposes [38, 59, 66], in some other studies, irradiation doses of ≥ 60 Gy improved OS [23, 37]. In an analysis of 1,288 patients with non‐resected ATC from the National Cancer Database (NCDB), Pezzi et al. document an improved 1‐year OS rate for Stage IVB and IVC patients who received 60 to 75 Gy, compared to those treated with less than 60 Gy [74]. Furthermore, Jonker et al. determined that RT dosages exceeding 50 Gy were more efficient than dosages below 50 Gy [64]. Generally, the results of included studies support that higher cumulative dosages of RT and EBRT are more beneficial than lower doses, even in unresectable tumors.

Currently, the most commonly utilized ChT treatments include those direct against cell division machinery (taxane, paclitaxel, or docetaxel), DNA repair pathways (anthracycline or doxorubicin), and DNA structure (cisplatin or carboplatin) [10]. Furthermore, the preferred application of cytotoxic chemotherapy is taxane monotherapy, with anthracyclines or platinum‐based agents added when necessary. Although combined treatment approaches consisting of surgery, RT, and ChT significantly prolonged median OS overall, ChT prescription was observed to provide different results in some studies. Wendler et al. observed that any form of systemic treatment (including weekly doxorubicin, paclitaxel, paclitaxel and pemetrexed, paclitaxel and carboplatin, doxorubicin and cisplatin, and tyrosine kinase inhibitors) was associated with prolonged OS in Stage IVC subjects. Likewise, the combination of paclitaxel and pemetrexed was statistically significantly correlated with a greater probability of longer OS compared to other regimens [59]. Kasemsiri et al. found that the combination of ChT with surgery is associated with more extended OS than pairing surgery and RT, while in the Brignardello et al. investigation, ChT administration was correlated with dismal outcomes [16, 65]. Further, some studies have reported that adding RT to surgery is significantly more effective than coupling surgery with ChT [48, 55].

Along with other treatment modalities, the use of molecular targeted therapy has been strongly recommended in the management of ATC, especially for those who have distinct mutations [79]. TKI drugs like sorafenib, pazopanib, and lenvatinib act by targeting Raf kinase mutations such as the *BRAF* ^*V600E*^ mutation arising in nearly 25–45% of ATC patients, receptor tyrosine kinases associated with angiogenesis such as vascular endothelial growth factor receptor (VEGFR) 1 ‐2 and ‐3, platelet‐derived growth factor receptor (PDGFR)‐β, and receptor tyrosine kinases associated with tumor progression (Flt‐3, c‐kit), respectively [73]. These targeted therapeutic agents have demonstrated promising outcomes in combination with each other, as well as different treatment modalities [73, 80]. Furthermore, combining the BRAF inhibitor dabrafenib with the MEK inhibitor trametinib demonstrated significant clinical activity, achieving a confirmed overall response rate of 69% in cases suffering from locally advanced or metastatic BRAF^V600E^–mutant ATC [81]. This combination therapy has been approved by the United States Federal Drug Agency (FDA) for the treatment of ATC with BRAF mutation [73]. Prior findings also support that combining surgery with a treatment regimen containing anti‐*BRAF* ^*V600E*^ mutation or lenvatinib resulted in improved OS in subjects with *BRAF* ^*V600E*^ mutation ATC [7, 21, 24]. Additionally, Vemurafenib is a small‐molecule drug that selectively inhibits oncogenic BRAF kinases, which has demonstrated an overall response rate of 29% of ATC cases [82]. RET inhibitors are another kind of targeted therapeutic agent that could be beneficial for RET‐altered thyroid cancers, such as ATC [83, 84]. Furthermore, lenvatinib/pembrolizumab has demonstrated promising results, as seen in the ATLEP trial, where 66% of ATC patients achieved complete remission, with median progression‐free survival of 17.75 months and OS of 18.5 months, particularly in those with high tumor mutational burden or programmed death ligand‐1expression (> 50%). However, toxicity led to dose modifications in nearly 50% of patients, underscoring the requirement for more precise and careful management [85]. Ongoing phase II trials aim to assess these approaches further, refine patient selection, and optimize treatment strategies.

Notably, the patient's characteristics and genomic landscape can significantly impact the disease prognosis. It has been claimed that older age, early distant metastatic disease, and a large primary tumor diameter (> 5 cm) negatively affect the prognosis of the disease [86]. A nationwide cohort study conducted in the Netherlands, encompassing 812 cases diagnosed with ATC from 1989 to 2016, reported an overall 1‐year survival rate of 12%, with a 1‐year survival rate of 21.6% observed in patients without distant metastases [87]. Prognostic factors associated with improved survival included being younger than 65 years, receiving multimodal treatment (involving more than two to three modalities), absence of distant metastases, and lack of bilateral lymph node involvement. Another investigation, which was performed in a tertiary academic hospital in the United States with 45 patients, determined that smaller tumor size was associated with improved survival [88]. Age, distant metastasis, and tumor size were also confirmed as independent prognostic factors for worse prognosis in another retrospective regional study [89]. In terms of genome alteration, ATC is nearly always fatal when a BRAF^V600E^ transgene is expressed concurrently with TP53 deletion and/or when both PTEN and TP53 are inactivated or mutational activation of PIK3CA occurs, which underlines the targeted therapy role in the management of ATC [90, 91].

In line with our study, Hu et al. systematic review results showed that patients who did not receive surgery, patients who underwent surgery only, and subjects who have employed surgery with following CRT exhibited a median OS of 2.1, 6.6, and 9.6 months, respectively [92]. They also concluded that primary surgery with maximal resection, as well as earlier stages of the disease, is associated with more favorable outcomes, consolidating the findings of previous studies about the importance of the type of surgery and disease condition on the outcome. Similarly, Oliinyk et al., in another systematic review, support the beneficial advantages of multimodality treatment with primary surgery, ChT, and RT, especially with a cumulative dosage of > 50 Gy [22]. Furthermore, they provide evidence that metastases have limitations in selecting the appropriate treatment. These findings were also supported by Kwon et al. in another systematic review, who confirmed the benefits of postoperative RT over surgery alone in terms of survival [93].

It is crucial to note that our study is based on the findings of multiple retrospective cohort studies, which introduces potential selection bias. Therefore, further research with neutralized bias and enhanced power is needed to validate these results and establish standardized treatment guidelines for ATC based on patient characteristics. Moreover, this systematic review was conducted through a qualitative assessment of previous studies; therefore, meta‐analysis studies are warranted to validate these results. In addition, various stages of ATC were included and evaluated in this study, which might have introduced substantial heterogeneity. Although many studies have evaluated different therapeutic options and prepared advantageous data to help specialists choose the most effective treatment approach for the patient, our study is the only one focusing on surgery because of its potential role as a curative choice in candidate patients with resectable tumor as well as for reaching locoregional control at IVC stage. Therefore, despite the limitations, our study provides valuable insights into the potential of surgical management and multimodal treatment approaches for improving the survival of ATC patients. Along with all the benefits of surgery, specialists should consider previous treatments, comorbidities, and patient characteristics to select the most suitable treatment method for individuals. Moreover, identifying specific surgical techniques in combination with CRT and targeted therapies may promote local control, as well as OS.

## 5 Conclusions

ATC is a progressive, lethal disease with no definitive treatment. Maximal resection surgery plus postoperative CRT may provide better survival outcomes in patients with stage IVA/B disease. In patients for whom surgery is not possible, such as IVC patients with distant or local metastasis, systemic therapy might be associated with superior locoregional control. Nonetheless, surgical resection, along with other recommended treatments, may also be considered in ATC stage IVC patients with a low burden of distant metastasis for locoregional disease control. In all stages, however, the characteristics of the patient can influence the outcome. ATC patients should be subjected to further multicenter trials to determine the most effective management.

## Author Contributions

**Hossein Negahban:** conceptualization, project administration, writing – review and editing, writing – original draft, Investigation. **Nazila Heidari:** writing – review and editing, writing – original draft, validation, investigation, data curation. **Amirhossein Heidari:** data curation, validation, investigation, writing – original draft, writing – review and editing. **Yekta Ghane:** data curation, validation, investigation, writing – original draft, writing – review and editing. **Mohammad Shirkhoda:** conceptualization, methodology, writing – review and editing. **Amirmohsen Jalaeefar:** conceptualization, methodology, project administration, writing – review and editing, supervision.

## Ethics Statement

The authors have nothing to report.

## Consent

The authors have nothing to report.

## Conflicts of Interest

The authors declare no conflicts of interest.

## 6 Transparency statement

Corresponding authors (Amirmohsen Jalaeefar and Mohammad Shirkhoda) affirm that this manuscript is an honest, accurate, and transparent account of the study being reported, that no important aspects of the study have been omitted and that any discrepancies from the study as planned (and, if relevant, registered) have been explained.

## Supporting information


**Table S1.** PRISMA 2020 checklist for reporting systematic.

**Table S2.** PRISMA 2020 for Abstracts Checklist.

**Table S3.** Comprehensive details of the search strategy of each database.

**Table S4.** The quality assessment of 56 studies was included in this systematic review.

## Data Availability

The data that supports the findings of this study are available in the supporting material of this article. The authors confirm that the data supporting the findings of this study are available within the article and its supporting materials.
