# Supplementary material for: Evaluation of Treatment Response and Survival Outcomes in Anaplastic Thyroid Cancer Patients Following Surgery With and Without Other Treatment Modalities: A Systematic Review
Source: Health Sci Rep. 2025 Apr 30;8(5):e70710. doi: 10.1002/hsr2.70710 (PMC12042218; doi:10.1002/hsr2.70710)
Supplement: Supplementary file 3 — Table S3. Comprehensive details of the search strategy of each database. [file HSR2-8-e70710-s002.docx]

**Table S3**. Comprehensive details of the search strategy of each database.

| **Database**  **(Search date)** | **Step** | **Search strategy** | **Number of results** |
| --- | --- | --- | --- |
| **PubMed**  **Jun 1th, 2023** | #1 | **(((((((((((("Anaplastic Thyroid Carcinoma"[Title/Abstract]) OR ("Anaplastic Thyroid Carcinomas"[Title/Abstract])) OR ("Carcinoma, Anaplastic Thyroid"[Title/Abstract])) OR ("Carcinomas, Anaplastic Thyroid"[Title/Abstract])) OR ("Thyroid Carcinomas, Anaplastic"[Title/Abstract])) OR ("Thyroid Cancer, Anaplastic"[Title/Abstract])) OR ("Anaplastic Thyroid Cancer"[Title/Abstract])) OR ("Anaplastic Thyroid Cancers"[Title/Abstract])) OR ("Cancer, Anaplastic Thyroid"[Title/Abstract])) OR ("Cancers, Anaplastic Thyroid"[Title/Abstract])) OR ("Thyroid Cancers, Anaplastic"[Title/Abstract])) OR ("Thyroid Carcinoma, Anaplastic"[Mesh])) OR ("undifferentiated thyroid carcinoma"[Title/Abstract])** | 678,532 |
|  | #2 | **(((("therapy"[Subheading] OR "** **therapeutics"[MeSH Terms]) OR ("therapy"[Title/Abstract])) OR ("therapeutics"[Title/Abstract])) OR ("treatment"[Title/Abstract])) OR ("treatment"[MeSH Terms])** | 13,148,811 |
|  | #3 | “Review”[Publication Type] OR “Review Literature as Topic”[MeSH] OR “Systematic Review”[Publication Type] OR “Systematic Reviews as Topic”[MeSH] OR “Meta-Analysis”[Publication Type] OR “Meta-Analysis as Topic”[MeSH] OR “Network Meta-Analysis”[MeSH] | 3,357,006 |
|  | #4 | #1 AND #2 NOT #3 filters: from 2000-2023 | 1,681 |
| **Scopus**  **Jun 1th, 2023** | #1 | ( TITLE-ABS-KEY ( "Anaplastic Thyroid Carcinoma" ) OR TITLE-ABS-KEY ( "Anaplastic Thyroid Carcinomas" ) OR TITLE-ABS-KEY ( "Carcinoma, Anaplastic Thyroid" ) OR TITLE-ABS-KEY ( "Carcinomas, Anaplastic Thyroid" ) OR TITLE-ABS-KEY ( "Thyroid Carcinomas, Anaplastic" ) OR TITLE-ABS-KEY ( "Thyroid Cancer, Anaplastic" ) OR TITLE-ABS-KEY ( "Anaplastic Thyroid Cancer" ) OR TITLE-ABS-KEY ( "Anaplastic Thyroid Cancers" ) OR TITLE-ABS-KEY ( "Cancer, Anaplastic Thyroid" ) OR TITLE-ABS-KEY ( "Cancers, Anaplastic Thyroid" ) OR TITLE-ABS-KEY ( "Thyroid Cancers, Anaplastic" ) OR TITLE-ABS-KEY ( "Thyroid Carcinoma, Anaplastic" ) OR TITLE-ABS-KEY ( "undifferentiated thyroid carcinoma" ) ) | 3,772 |
|  | #2 | ( TITLE-ABS-KEY ( "therapy" ) OR TITLE-ABS-KEY ( "therapeutics" ) OR TITLE-ABS-KEY ( "therapeutic" ) OR TITLE-ABS-KEY ( "treatment" ) OR TITLE-ABS-KEY ( "treatments" ) AND TITLE-ABS-KEY ( "therapies" ) ) | 5,260,952 |
|  | #3 | ( TITLE-ABS-KEY ( "Review" )  OR  TITLE-ABS-KEY ( "Review Literature as Topic" )  OR  TITLE-ABS-KEY ( "Systematic Review" )  OR  TITLE-ABS-KEY ( "Systematic Reviews as Topic" )  OR  TITLE-ABS-KEY ( "Meta-Analysis" )  OR  TITLE-ABS-KEY ( "Meta-Analysis as Topic" )  OR  TITLE-ABS-KEY ( "Network Meta-Analysis" ) ) | 6,016,357 |
|  | #4 | #1 AND #2 NOT #3 filters: from 2000-2023 | 1047 |
| **Web of Science**  **Jun 1th, 2023** | #1 | "Anaplastic Thyroid Carcinoma" (Topic) OR "Anaplastic Thyroid Carcinomas" (Topic) OR "Carcinoma, Anaplastic Thyroid" (Topic) OR "Carcinomas, Anaplastic Thyroid" (Topic) OR "Thyroid Carcinomas, Anaplastic" (Topic) OR "Thyroid Cancer, Anaplastic" (Topic) OR "Anaplastic Thyroid Cancer" (Topic) OR "Anaplastic Thyroid Cancers" (Topic) OR "Cancer, Anaplastic Thyroid" (Topic) OR "Cancers, Anaplastic Thyroid" (Topic) OR "Thyroid Cancers, Anaplastic" (Topic) OR "Thyroid Carcinoma, Anaplastic" (Topic) OR "undifferentiated thyroid carcinoma" (Topic) | 3,080 |
|  | #2 | (((((TS=("therapy")) OR TS=("therapies" )) OR TS=("therapeutic")) OR TS=("therapeutics")) OR TS=("treatment")) OR TS=("treatments") | 8,498,944 |
|  | #3 | **((((((TS=("Review")) OR TS=("Review Literature as Topic")) OR TS=("Systematic Review")) OR TS=("Systematic Reviews as Topic")) OR TS=("Meta-Analysis")) OR TS=("Meta-Analysis as Topic")) OR TS=("Network Meta-Analysis")** | 2,957,957 |
|  | #4 | #1 AND #2 NOT #3 | 1,589 |
| **Total: 4317** | | | |
